# New Insights Into the Role of Autophagy in Liver Surgery in the Setting of Metabolic Syndrome and Related Diseases

**DOI:** 10.3389/fcell.2021.670273

**Published:** 2021-06-01

**Authors:** Ana Isabel Álvarez-Mercado, Carlos Rojano-Alfonso, Marc Micó-Carnero, Albert Caballeria-Casals, Carmen Peralta, Araní Casillas-Ramírez

**Affiliations:** ^1^Department of Biochemistry and Molecular Biology II, School of Pharmacy, Granada, Spain; ^2^Institute of Nutrition and Food Technology “José Mataix”, Biomedical Research Center, Parque Tecnológico Ciencias de la Salud, Granada, Spain; ^3^Instituto de Investigación Biosanitaria ibs. GRANADA, Complejo Hospitalario Universitario de Granada, Granada, Spain; ^4^Institut d’Investigacions Biomèdiques August Pi i Sunyer (IDIBAPS), Barcelona, Spain; ^5^Hospital Regional de Alta Especialidad de Ciudad Victoria “Bicentenario 2010”, Ciudad Victoria, Mexico; ^6^Facultad de Medicina e Ingeniería en Sistemas Computacionales de Matamoros, Universidad Autónoma de Tamaulipas, Matamoros, Mexico

**Keywords:** autophagy, metabolic syndrome, ischemia-reperfusion, liver surgery, transplantation

## Abstract

Visceral obesity is an important component of metabolic syndrome, a cluster of diseases that also includes diabetes and insulin resistance. A combination of these metabolic disorders damages liver function, which manifests as non-alcoholic fatty liver disease (NAFLD). NAFLD is a common cause of abnormal liver function, and numerous studies have established the enormously deleterious role of hepatic steatosis in ischemia-reperfusion (I/R) injury that inevitably occurs in both liver resection and transplantation. Thus, steatotic livers exhibit a higher frequency of post-surgical complications after hepatectomy, and using liver grafts from donors with NAFLD is associated with an increased risk of post-surgical morbidity and mortality in the recipient. Diabetes, another MetS-related metabolic disorder, also worsens hepatic I/R injury, and similar to NAFLD, diabetes is associated with a poor prognosis after liver surgery. Due to the large increase in the prevalence of MetS, NAFLD, and diabetes, their association is frequent in the population and therefore, in patients requiring liver resection and in potential liver graft donors. This scenario requires advancement in therapies to improve postoperative results in patients suffering from metabolic diseases and undergoing liver surgery; and in this sense, the bases for designing therapeutic strategies are in-depth knowledge about the molecular signaling pathways underlying the effects of MetS-related diseases and I/R injury on liver tissue. A common denominator in all these diseases is autophagy. In fact, in the context of obesity, autophagy is profoundly diminished in hepatocytes and alters mitochondrial functions in the liver. In insulin resistance conditions, there is a suppression of autophagy in the liver, which is associated with the accumulation of lipids, being this is a risk factor for NAFLD. Also, oxidative stress occurring in hepatic I/R injury promotes autophagy. The present review aims to shed some light on the role of autophagy in livers undergoing surgery and also suffering from metabolic diseases, which may lead to the discovery of effective therapeutic targets that could be translated from laboratory to clinical practice, to improve postoperative results of liver surgeries when performed in the presence of one or more metabolic diseases.

## Introduction

Obesity is the major risk for the development of metabolic syndrome (MetS), a prevalent entity in Western societies that includes cardiovascular risk factors i.e., hypertension, diabetes mellitus, and, insulin resistance (Nakao et al., 2012). The combination of these metabolic disorders compromises liver function, manifested as non-alcoholic fatty liver disease (NAFLD), a risk marker in type 2 diabetes (T2D) and MetS. NAFLD compromises pre-existing hepatic disease and is progressively on the rise throughout the world (Gadiparthi et al., 2020). Albeit controversial, the use of fatty livers in liver transplantation is also increasing due to the paucity of available organs, but they tend to poorly tolerate the ischemia/reperfusion (I/R) process, leading to primary non-function, early graft dysfunction, and graft loss. Significant steatosis has also been more frequent in tumor-associated hepatic resections in which I/R is also sub-par, and carries high morbidity in the immediate postoperative period (Micó-Carnero et al., 2021).

The complex molecular interrelation between obesity, MetS, insulin resistance, and diabetes does not allow us to clarify which is the precise concatenation of events driving NAFLD. However, a common denominator in all of such metabolic diseases is autophagy. Abnormal autophagy fails to restore homeostasis, promotes the development of obesity-associated diseases, and increases insulin resistance (Yang et al., 2010). The pathophysiology of diabetes mellitus types I and II, as well as β-cell dysfunction, has been associated with abnormal autophagy mechanisms (Gonzalez et al., 2011). Abnormal autophagy may also underlie I/R injury and NAFLD in partially resected or transplanted livers (Zhao et al., 2016). Moderate I/R injury causes cell dysfunction by autophagy and activates survival recovery systems, but prolonged I/R-mediated cell damage may lead to apoptosis and necrosis (Lopez-Neblina et al., 2005; Zhu J. et al., 2016; Ling et al., 2017). Furthermore, hyperglycemia exacerbates liver ischemic injury (Behrends et al., 2010). Patients with diabetes and requiring a liver resection are at great risk of hepatic injury during surgery (Han et al., 2014), and this also applies to potential liver transplant diabetic donors. Due to the great increase in the prevalence of NAFLD and diabetes, their association is frequently present in society and hence, in potential liver graft donors, and patients requiring a hepatic resection. This scenario requires the development of effective strategies to improve post-operative results in NAFLD and/or diabetic patients subjected to liver surgery; hence, a thorough understanding of the signaling pathways in NAFLD, MetS, diabetes, insulin resistance, and I/R injury are paramount.

Based on the previously presented challenges, we will specifically review the effects of obesity, MetS, insulin resistance, and/or diabetes in the postoperative outcomes from liver resection and transplantation, and the crucial role of autophagy in that setting. A better understanding of the molecular mechanisms related to autophagy underlying such conditions is extremely relevant to develop effective therapeutic targets to improve the post-operative outcomes of hepatic surgeries when associated with the mentioned comorbidities. In this sense, it is important the knowledge whether the autophagy process is different in livers affected by metabolic comorbidities with and without surgical intervention. Then, we will initially analyze the effects of MetS and related diseases as obesity, insulin resistance, NAFLD, and/or diabetes on livers without surgery, and the involvement of the autophagy process in such context. We will then focus on how metabolic diseases negatively affect hepatic I/R injury inherent to liver surgery. Finally, we will present the mechanisms underlying the role of autophagy in damage in surgically intervened livers, especially those suffering metabolic diseases.

## Involvement of Hepatic Autophagy in the Effects of Metabolic Dysfunctions on the Liver

Autophagy is a vacuolar self-digestion, whereby intracellular proteins, fatty acids (FA), organelles, and cellular detritus (cargo) are degraded by lysosomal enzymes and recycled into their basic units, to be reused in the cytoplasm for survival, differentiation, etc. (Mizushima et al., 2010; Miyamoto and Heller, 2016). Autophagy can be selective or non-selective since this cellular response can be directed to degrade a specific organelle (i.e., mitophagy or lipophagy) (Kim et al., 2016). Autophagy begins with the development and elongation of a membrane, the phagophore that in turn, is transformed into a vesicle surrounded by two membranes in which the cargo is isolated. Microtubule-associated protein light-chain 3 (LC3) participates in the formation of autophagosomes; its cleavage by autophagy gen (Atg)4B results in LC3-I, which conjugates with phosphatidylethanolamine via two consecutive reactions catalyzed by the E1-like enzyme Atg7, and the E2-like enzyme Atg3, thus forming lipidated LC3-II associated with the autophagosomal membrane. The autophagosome matures by fusing with lysosomes to create autophagolysosomes where its selected cargo is degraded (Kabeya et al., 2000; Kirisako et al., 2000). The p62/sequestosome (SQSTM) is a ubiquitin-binding protein that recognizes ubiquitinated cargo and links with autophagosomes through direct interaction with LC3-II (Lamark et al., 2003; Pankiv et al., 2007). Since LC3-II and p62 are both degraded in the autolysosome with autophagic cargo, accumulation of LC3-II and p62 is regarded as a robust marker of impaired autophagic flux (Lee et al., 2012). Lysosome-associated membrane proteins (LAMPs) are essential for autophagosome-lysosome fusion during autophagy and are responsible for lysosomal proteolytic activity. When LAMP1 and LAMP2 are inhibited, then autophagosome and lysosome fusion is inhibited, and this is associated with the accumulation of LC3-II and p62 suggesting a decrease in autophagy flux (Fortunato et al., 2009). Lysosomal proteases, such as cathepsin, also play a key role in autolysosome degradation (Uchiyama, 2001), and therefore, lysosome dysfunction may be involved with autophagic flux impairment. In line with this, defective cathepsin B, cathepsin D, and cathepsin L enzyme activity resulted in impaired lysosomal acidification and this occurred concomitantly with autophagic flux blockade, including autophagosome accumulation and decreased degradation of SQSTM1/p62 (Mareninova et al., 2009). Considering all autophagy stages, there exists an updated consensus that suggests that the real status of autophagy should be assessed not only by the number of autophagosomes and autolysosomes but also by evaluating the actual autophagic flux, such as monitoring the clearance of cell components in autolysosomes (du Toit et al., 2018).

The liver is rich in lysosomes and has a high level of stress-induced autophagy. In fact, reactive oxygen species (ROS) give rise to lysosomal dysfunction and autophagy flux impairment, avoiding the correct degradation of damaged cellular components (Jung et al., 2020). In addition, enhanced endoplasmic reticulum (ER) stress can deregulate lysosomal acidification and thus, blocking autophagy in hepatocytes (Chen et al., 2019a). This provokes hepatotoxicity, cell death, and alteration of hepatic function (Jung et al., 2020). A close association between autophagy functionality, obesity, and liver disease has been posited (He et al., 2016), and consequently, the understanding of the molecular mechanisms underlying this relationship occurring in the liver may be the basis for the design of new protective strategies for livers affected by metabolic diseases and undergoing a liver surgery process. The vast majority of reviews that address the role of autophagy in obesity, MetS, insulin resistance, NAFLD or diabetes, does not clearly distinguish which are the molecular signaling pathways related to autophagy that come about at the liver tissue. Therefore, the findings that have been reported about the effects of such metabolic diseases on the hepatic autophagy process are presented below.

### Metabolic Syndrome and Associated Disorders

Although several studies have attempted to determine the origin of the MetS, due to its complexity, a clear etiology remains to be established. MetS refers to central obesity, insulin resistance, impaired glucose tolerance, dyslipidemia, and elevated blood pressure (Simmons et al., 2010). Excessive fat accumulation in obesity associated with the MetS and its macrovascular complications activates the immune system and chronic states of low-grade inflammation. Altered signaling at the molecular level in adipose tissue, negatively affects the liver as causes hepatic infiltration by macrophages and other immune cells (Esser et al., 2014). This event is not the only one that damages liver tissue. Once adipose tissue has reached its storage capacity, excess calories are redirected to other depots and lead to ectopic fat accumulation. Visceral fat drains into the portal vein, and then the liver is targeted by multiple metabolites and adipokines (Schleinitz et al., 2014). A connection between obesity, progressive lipid accumulation in the liver, and T2D in humans has been established (Goto et al., 2017). Diabetes is linked particularly to NAFLD, and indeed, up to 70% of diabetic patients develop NAFLD (Williamson et al., 2011). Hepatic lipids tend to further exacerbate insulin resistance by interfering with insulin signaling, thus perpetuating the vicious cycle. In fact, FA increases gluconeogenesis, lipogenesis, and chronic inflammation, which foster hepatic glucose production, resulting in hyperglycemia and hepatic insulin resistance (Pereira et al., 2015; Wang Z. et al., 2017).

Based on evidence obtained from *in vivo* and *in vitro* studies, autophagy has been suggested to underlie the pathophysiology of MetS, obesity, insulin resistance, and diabetes in the liver (Ogihara et al., 2002; Engeli et al., 2003; Yang et al., 2010; Putnam et al., 2012; Wang H.J. et al., 2015). Whether autophagy is a protective factor against obesity or a manifestation of impaired adipose tissue function, remains to be determined. Obesity is often associated with liver steatosis and insulin resistance. In obesity, autophagy decreases in hepatocytes, and metabolism is impaired. The release of lipids stored in droplets is mediated by autophagy; if inhibited, promotes fatty liver development. Mice with the hepatocyte-specific Atg7 deletion, develop fat droplets, whereas Atg7 reestablishment ameliorates hepatic function. Yang et al. described lower protein levels of Atg7, Beclin 1 (Atg6), LC3, Atg5, and elevated p62 in the livers of obese mice (Yang et al., 2010). Concerning other metabolic disorders, in rats with MetS increased hepatic autophagy activity manifested by elevated LC3-II/I, Beclin-1, mammalian target of rapamycin (mTOR), and p62 autophagy-related proteins, as well as phosphorylated adenosine 5′-monophosphate-activated protein kinase (AMPK) down-regulation, has been reported (Cui et al., 2020). These results appear to reflect an opposite role for autophagy in obesity and MetS, however, they must be carefully analyzed. Since an increase in autophagy-dependent protein expression can result from one of two mechanisms, it could reflect either an increase in autophagosome synthesis or an arrest in degradation.

Accumulation of autophagosomes in the liver in the setting of obesity has been demonstrated, and to explain this, a study carried out with a genetic obese experimental model found that a defect in lysosomal acidification and proteinase activity of cathepsin impaired hepatic autophagic degradation (Inami et al., 2011). On the other hand, an investigation performed with a diet-induced obese model indicated that impairment of autophagic flux was due to blockage of autophagosome-lysosome fusion, without alteration in lysosomal environment, including acidification and hydrolytic function; and this was associated with ER stress (Miyagawa et al., 2016). These results would indicate that possibly, different experimental models could induce variations in some autophagy signaling pathways. The autophagosome-lysosome degradation process plays a role in the pathogenesis of obesity-mediated diabetes. In mice, deficiencies in LAMP2, which is crucial in the fusion and degradation of autophagosomes with lysosomes, prevent the development of high-fat diet (HFD)-induced obese T2D and increases energy expenditure, in turn, associated with hepatic fibroblast growth factor 21 (FGF21) overproduction. The expression of ER stress-related proteins was increased in the liver of HFD-fed LAMP2-deficient mice and it was suggested that ER stress was involved in the hepatic induction of FGF21 (Yasuda-Yamahara et al., 2015). In line with these results, it has been also reported that insufficient autophagosome formation in the liver induced mitochondrial stress, increased ATF4-FGF21 pathway activity, and protected from diet-induced obesity and insulin resistance (Kim et al., 2013).

In the context of metabolic diseases, autophagy is a dynamic process in the liver that changes as a function of time. Experimental studies demonstrate an increased hepatic autophagy activity in HFD-induced obesity and hepatic steatosis. However, the raise of HFD-induced autophagy only lasts a few weeks and in fact, it decreases in chronic obesity due to cellular stress. In this sense, it has been observed that autophagy remains active for 7 weeks under HFD conditions, but disappears after the eighth week (Adkins et al., 2013). These results should be cautiously interpreted. Although in several investigations experimental results may indicate an increased expression of autophagy markers and autophagosome number, in such studies the role and values of autophagic flux were not appropriately determined (the total process of autophagosome synthesis, substrate delivery, and lysosomal degradation) (Menikdiwela et al., 2020). Thus, it is not possible to ascertain whether such results are completely reliable as evidence of increased autophagic activity.

Several factors may regulate hepatic autophagy in MetS and related disorders. Hepatic ER stress suppresses autophagy in high-fat-fed mice and *ob/ob* mice, and disruption of autophagy function by genetic ablation of Atg proteins can lead to ER stress (Choi et al., 2018). Collectively, these data suggest that hepatic ER stress is directly related to the suppression of autophagy. In genetic and in diet-induced mouse models of obesity, lower levels of hepatic autophagy and increased ER stress with concomitant insulin resistance, appear to follow a pattern. Decreased autophagy led to greater ER stress and insulin resistance in murine hepatocytes (Menikdiwela et al., 2020). ER stress and increased lipogenesis are potential mechanisms accounting for insulin resistance mediated by high fructose feedings. Higher levels of calpain 2 in hepatocytes decrease autophagy in obese models, while its inhibition increases it; melanocortin 3 receptor (MC3R) also regulates hepatic autophagy by possibly acting on transcription factor EB (TFEB) signaling. Another mechanism that decreases hepatic autophagy is via the forkhead box O (FOXO) transcriptional factor. FOXO is a key regulator of vacuolar protein sorting 34 (Vps34) and Atg12, responsible for autophagy initiation. Its activity is suppressed by increased insulin levels and serine-threonine protein kinase Akt (Akt) activation, and hence, autophagy decreases in MetS (Liu et al., 2009). Nutrients and insulin also regulate the autophagy process in the liver, since they are two powerful suppressors of autophagy (Vanhorebeek et al., 2011). Regarding the effects of some nutrients, hepatic autophagy was suppressed in the presence of ER stress after mice were fed high fructose diets for 2–12 h, 2 weeks, as well as in liver explants incubated with fructose medium (Wang H. et al., 2015). Increased secretion of insulin by β-cells decreases hepatic autophagy and promotes insulin resistance in hepatocytes (Quan et al., 2012). In line with this, in MetS hyperinsulinemia up-regulates mammalian target of rapamycin complex 1 (mTORC1) activity and suppresses hepatic autophagy. Concurrently, activated hepatic mTORC1 further phosphorylates the insulin receptor through ribosome S6 protein kinase 1 (S6K1), and this leads to insulin resistance (Wang et al., 2012). As regards hyperglycemia and diabetes, hepatic autophagy comes to play an important role in glucose homeostasis (Wang H.J. et al., 2015). Cryptochrome 1 (CRY1) decreases hepatic glucose production, and its timely removal by autophagy promotes glucose production. Obesity increases CRY1 degradation by autophagy, thus increasing glucose production and circulating blood sugar levels (Toledo et al., 2018).

Whether defective autophagy is cause or consequence of hepatic insulin resistance remains to be determined. This distinction might be pivotal when managing diabetic patients undergoing liver surgery. For instance, if defective autophagy induces insulin resistance, a promising strategy for such patients may be to treat hyperglycemia with short-term intensive insulin therapy, since autophagy is already defective, and this treatment has proven effective in the control of glucose levels (Kramer et al., 2013). However, if an increase in insulin results in decreased hepatic autophagy, insulin therapy may render the liver vulnerable to other stressors. In diabetes conditions, research indicates that there is an upregulation of autophagy and therefore, a decrease in autophagy could be useful in hepatic glucose homeostasis regulation, and to a certain extent, prevent the development of diabetes and its complications. Although these findings contribute to a better understanding of the role of autophagy in the liver affected by diabetes, there is still very little literature on the molecular events underlying hepatic autophagy in diabetic conditions, indicating that more research is required in this regard.

### NAFLD

Non-alcoholic fatty liver disease is the most common cause of abnormal liver function and it covers a broad spectrum of liver changes, ranging from simple steatosis to non-alcoholic steatohepatitis (NASH), liver cirrhosis, and hepatocellular carcinoma (Zhang et al., 2018). The prevalence of NAFLD is on the rise throughout the Western world since it is strongly associated with obesity, insulin resistance, MetS, and T2D. In fact, NAFLD is generally considered to be the hepatic manifestation of MetS, and research has confirmed that hepatic lipid accumulation is responsible for hepatic insulin resistance and whole-body insulin resistance. Lee et al. have reported that obese adolescents with fatty liver are at greater risk of developing systemic insulin resistance than those with a healthy liver (Lee et al., 2015). Thus, NAFLD has become a major public health issue, with potentially serious consequences (Parafati et al., 2015). In this sense, NAFLD is rapidly becoming the leading indication for liver transplantation (Zhang et al., 2018).

A consensus has been reached, suggesting that autophagy plays a crucial role in the pathogenesis of NAFLD, and in fact, several studies have indicated impaired autophagy (Samala et al., 2017). Autophagy is inhibited in the livers of NAFLD and NASH murine models. Furthermore, enhancing autophagy by overexpressing Atg7, improved hepatic steatosis in ob/ob mice and mice fed a HFD (Gong et al., 2016). Increased levels of LC3-II and p62 reflect a defect in the autophagic flux (a failure in the clearance of autophagosomes), and this has been observed in the liver of C57BL/6 mice when fed a HFD. Similar results have been reported in mice fed a methionine/choline-deficient (MCD) diet that is usually used to induce NASH (Wang et al., 2018). Also, autophagy markers, LC3-II and p62, were impaired in the liver of genetically leptin-deficient ob/ob mice that are obese and insulin resistant, and that develop NAFLD like that observed in humans (Yang et al., 2010). The presented body of evidence suggests that under these conditions, clearance of autophagosomes is impaired. In NAFLD and NASH patients, p62 and LC3-II levels are increased and reflect autophagic flux impairment in the diseased livers (González-Rodríguez et al., 2014). Regardless, normal p62 values have also been reported in NAFLD and NASH patient livers (Lee et al., 2017). This issue remains a controversial subject. In the context of these metabolic diseases, a deficient autophagic flux is related to lysosome dysfunction. Autolysosome acidification is critical to the degradation of autophagosomal cargos for the maintenance of autophagic flux. In NASH, the precursor form of cathepsin D, an enzyme cleaved to its mature form upon acidification and involved in lysosome-dependent proteolysis, showed accumulation. In addition, the number of acidic lysosomes was reduced in steatotic hepatocytes. These events could be associated with the increased synthesis of asparagine by asparagine synthetase (ASNS). Expression of ASNS was elevated in steatohepatitis and knockdown of ASNS restored autophagic flux in MCD medium–cultured hepatocyte cell lines, as evidenced by the decreased accumulation of p62. As well, asparagine exposure in hepatocytes directly inhibited lysosome acidification, as evidenced by the failed cleavage of procathepsin D to its mature form, and the reduced number of acidic autolysosomes (Wang et al., 2018). In addition, to defects in acidification of lysosomes, other causes of lysosomal dysfunction related to worsened autophagic flux have been described in NAFLD-NASH. Chemokine (C-X-C motif) receptor 3 (CXCR3) inhibited autophagic flux in steatohepatitis since ablation of CXCR3 reduced p62 and LC3-II accumulation and ameliorated steatohepatitis. In this context, CXCR3 induced LAMP1 and LAMP2, which are membrane proteins crucial in autophagosome-lysosome fusion and proteolytic activity, indicating lysosome storage disorder (Zhang et al., 2016). According to what happens in other diseases, upregulation of LAMP might suggest an increase in the overall lysosomal mass that could be interpreted as an attempt to counteract lysosomal dysfunction (Houben et al., 2017). Last but not least, an increment in intracellular lipids, as occurs in NAFLD-NASH, altered the intracellular membrane lipid composition of both autophagosomes and lysosomes. This reduced the ability of autophagosomes to fuse with lysosomes and led to a decrease in autophagic flux (Koga et al., 2010).

**FIGURE 1:**
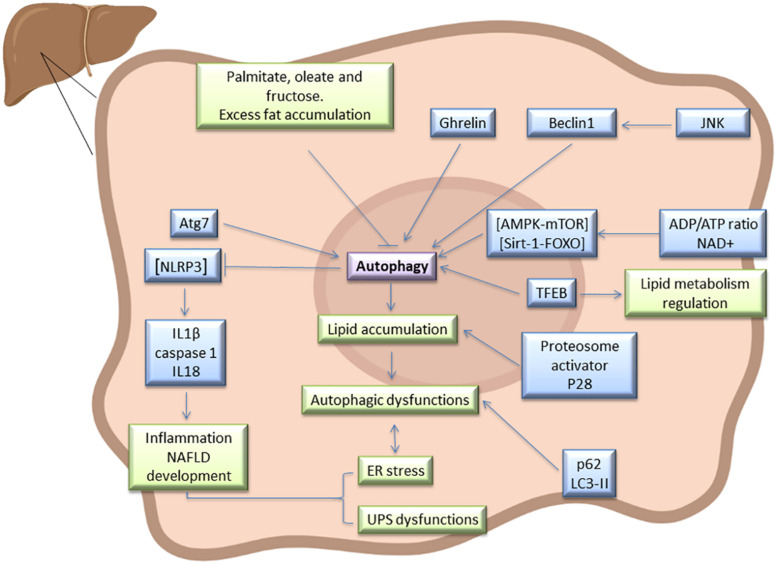
Autophagy signaling pathway in steatotic liver. Atg7, Beclin1, LC3-II, and p62 has been described as markers of autophagy in fatty liver, and this process is regulated by AMPK-mTOR, Sirt1-FOXO, and TFEB. In this type of liver, autophagy is interrelated with UPS, ER stress, JNK, ghrelin, and NLRP3 inflammasome signaling pathways, among others. As a result of interaction between all these mediators, dysfunction in lipid accumulation and inflammation may occurs in steatotic liver. Some diet components such as excess fat, palmitate, oleate or fructose could alter hepatic autophagy in steatotic livers and therefore affect NAFLD progression.

Hepatic ER stress and autophagy dysfunction increase with the onset of NAFLD in patients and dietary-induced obese models, whereas suppressing ER stress and activating autophagy ameliorate high-fat-induced hepatocyte apoptosis (Wang H. et al., 2015). NAFLD development requires additional diverse signaling pathways and mediators. In steatotic livers, autophagy is mainly regulated by the AMPK-mTOR and silencing information regulator 1 (Sirt1)-FOXO pathways, which are activated by increased adenosine diphosphate (ADP)/adenosine triphosphate (ATP) ratios and nicotinamide adenine dinucleotide (NAD +), respectively (Figure 1). Several studies suggest that AMPK activation or Sirt1 induction restore impaired autophagy and ameliorate hepatic lipid accumulation (Huang et al., 2017). The TFEB is another autophagy regulator that positively modulates lipid catabolism, as a result of the direct induction of the “coordinated lysosomal expression and regulation” (CLEAR) network, which includes genes that control autophagy, lysosome biogenesis, and lipolysis (Wang Z. et al., 2017). Interestingly, TFEB overexpression in mice in which autophagy was genetically suppressed by deletion of hepatic Atg7, did not preclude the development of or decrease fatty infiltration in hepatic steatosis, suggesting that the effects of TFEB on lipid metabolism require a functional autophagic pathway (Settembre et al., 2013). The induction of autophagy is dependent upon nutrient conditions, whereby in nutrient-rich conditions, mTOR inhibits the initiation of autophagy. Perhaps as a result of over-nutrition, mTOR signaling is frequently hyperactivated in the livers of obese mice and also suppresses TFEB activity (Singh et al., 2009). Hepatic steatosis improved in HFD-induced NAFLD, after mTOR inhibitor treatment, rapamycin (Gong et al., 2016). Autophagy is also dependent on c-Jun NH(2)-terminal kinase (JNK) whereby JNK activation contributes to Beclin-1 expression and modulates autophagy. Inhibition of JNK suppresses autophagy and decreases insulin resistance in NAFLD (Yan et al., 2017). Different ghrelin isoforms might upregulate autophagy and ameliorate liver disease, as proven in preclinical and clinical studies. It appears that acyl ghrelin up-regulation stimulates hepatic autophagy by suppressing tumor necrosis factor alpha (TNF-α) production via AMPK/mTOR. This protective mechanism leads to NAFLD improvement (Chorny et al., 2008). However, in experimental acute hepatitis and liver fibrosis, ghrelin administration decreases hepatic autophagy (Mao et al., 2015). These different ghrelin effects may result from differences in experimental conditions, drug doses, and cellular physiological states.

Dietary nutrients could affect autophagy in the liver and consequently regulate NAFLD progression. Under starvation, active autophagy leads to lipolysis and free FA production, providing additional energy sources. In conditions of excessive fat accumulation in the liver, autophagy is downregulated, leading to an additional increase in lipid accumulation in the liver, as it is observed in the liver of mice fed a HFD (Samala et al., 2017). Palmitate (one of the most abundant FA in the plasma of humans and rodents) is poorly converted into triglyceride-enriched lipid droplets (LD), and inhibits autophagy by inducing the caspase-dependent cleavage of Beclin1; its presence also induces a degree of lipid-induced apoptosis (lipoapoptosis) in hepatocytes (Ricchi et al., 2009). On the other hand, monounsaturated FA, such as oleate, stimulate the formation of triglyceride-enriched LD and induce autophagy but hardly affects lipoapoptosis. The formation of LD and the induction of autophagy are known to be protective mechanisms against saturated FA-induced lipotoxicity (Mei et al., 2011). These data indicate that not only excessive nutrition *per se* but also diet composition, are crucial factors fomenting NAFLD. Long-lasting intake of certain nutrients can also modulate hepatic autophagy. After 4 weeks of consuming a HFD, autophagy was activated to protect hepatocytes from lipotoxicity; further, apoptosis was activated after a HFD intake for 8 weeks, and autophagy initiation was suppressed. After 16 weeks on the HFD, an increase in the LC3II/LC3I ratio was demonstrated, suggesting that functional autophagy was disrupted after HFD consumption lasting more than 8 weeks (Hsu et al., 2016). A high fructose diet also suppresses liver autophagy and appears to be mediated by the activation of mTOR. High fructose-induced suppression of autophagy may cause ER stress, and the resulting changes in JNK/inhibitor of NF-κB kinase (IKK) and insulin signaling cascades. When autophagy is restored with pharmacological agents, ER stress is improved and all associated injurious events caused by a high fructose diet are corrected (Wang H. et al., 2015).

#### Lipophagy, a Form of Selective Autophagy, Is Significant in NAFLD

Lipophagy selectively degrades LD which are incorporated in vesicles, transported to lysosomes, and finally degraded into FA. This lipid degradation pathway in hepatocytes explains their ability to rapidly mobilize large amounts of lipids despite low levels of cytosolic lipases when compared with adipocytes (Zhang et al., 2018). In fact, sustained abnormalities in lipophagy in hepatocytes might be the basis of liver steatosis and steatohepatitis (Schott et al., 2019). Continuing studies in humans or animals have established that lipophagy contributes to the NAFLD development (Schulze and McNiven, 2014; Xiong et al., 2016; Chen et al., 2017b). As blood lipids infiltrate the liver, upregulation of the biogenesis of LD acts as a defense mechanism against toxic FA that are esterified into triglycerides and stored in LD. Pharmacological or genetic inhibition of autophagy increases triglyceride and cholesterol contents, and LD number and size, in hepatocytes treated with free FA and in mice fed a HFD. On the opposite, enhancing autophagy by Atg7 overexpression improved hepatic steatosis and insulin resistance in ob/ob mice and mice fed a HFD (Jeong Kim et al., 2017). Thus, this emerging evidence points that lipophagy could be a therapeutic target in NAFLD.

In the liver, lipophagy is mediated by complex transcriptional regulators of autophagy genes and is inhibited by the insulin and amino acid-mTOR signaling pathway via short- and long-term regulation mechanisms. Short-term inhibition is dependent on the mTOR complex, while long-term regulation is mediated by the transcription factors FOXO and TFEB (Stayrook et al., 2005; Oberkofler et al., 2010). Insulin receptor activation induces *de novo* lipogenesis and prevents autophagy-mediated LD degradation (Bechmann et al., 2012). Other regulators of lipophagy in the liver have been described. Oxidative stress appears to affect lipophagy, in fact, LD accumulated more readily in the liver in SOD1-knockout mice compared with the wild-type mouse, under fasting conditions. Lipophagy was abolished by oxidative stress, and lipoprotein secretion was suppressed in superoxide dismutase (SOD)1 deficiency, ultimately leading to LD accumulation (Kurahashi et al., 2015). Further, ROS increases perilipin 2 (PLIN2), which induces LD accumulation by blocking autophagy and negatively affecting droplet breakdown (Jin et al., 2018). Therefore, hepatic autophagy induction might modulate LD accumulation and ROS generation. Immunity-related guanosine triphosphatase family M (IRGM) is another key gene involved in lipophagy regulation, as IRGM knockdown inhibited autophagy and increased LD content in HepG2 and PLC/PRF/5 cells, and this process could be reversed by rapamycin (Lin et al., 2016). FGF21 can ameliorate NAFLD, partly re-establish insulin sensitivity, and correct several metabolic parameters through lipophagy in HepG2 cells overloaded with lipids (Zhu S. et al., 2016). The run domain beclin-1-interacting and cysteine-rich domain-containing protein (Rubicon) inhibit late-stage autophagy during the autophagosome-lysosome fusion. In livers with impaired Rubicon expression, ER stress and the accumulation of LD decreased, and also autophagy impairment is attenuated, thus indicating that Rubicon could abolish lipophagy, and hence cause the deposit of LD in the liver (Tanaka et al., 2016).

Enhancing lipophagy clears hepatocellular LD and this could drive to a strategy to mobilize hepatic lipids and prevent human NAFLD. An important issue to ponder is the possible cooperation or synergy between lipolysis and lipophagy in the regulation of hepatic lipid metabolism. Lipophagy may degrade larger LD into smaller droplets, which in turn increases LD surface area upon which cytosolic lipases can exert their action (Zhang et al., 2018). Recognizing lipophagy as an elementary process regulating lipid clearance in the liver enlightens new possibilities to design therapies that promote hepatic lipophagy and improve metabolic diseases.

#### Mitophagy and NAFLD

Mitochondrial autophagy (mitophagy) is a mitochondrial quality control process that degrades damaged mitochondrion and suppresses their production of ROS that might lead to mitochondrial dysfunction (Adeva-Andany et al., 2019). Hepatic FA accumulation can cause damaged mitochondria accumulation, which in turn, hinders mitochondrial respiratory chain function, and FA oxidative degradation. The accumulation of dysfunctional mitochondria leads to BCL2 and adenovirus E1B 19-kDa-interacting protein 3 (BNIP3)/BNIP3-like (BNIP3L, also known as NIX) (NIX)-mediated mitophagy, clearance of mitochondrial debris, and the reestablishment of mitochondrial function. Phosphatase and tensin homolog (PTEN)-induced putative kinase 1 (PINK1)/Parkin is another molecular mechanism that could be involved in hepatic mitophagy (Sato and Furuya, 2017).

Studies on mitophagy in NAFLD and related metabolic comorbidities are still scarce, however, they show that this molecular pathway could have broad therapeutic possibilities. Impaired mitophagy promotes macrophage infiltration that activates mitogen-activated protein kinase (MAPK) pathway, and causes inflammation, impairing mitochondrial quality control, and fostering the development of insulin resistance and hepatic steatosis. It has been demonstrated that stimulating mitophagy can inhibit hepatic lipid accumulation and temper insulin resistance (Su et al., 2019). In an experimental model of NAFLD, abnormal PINK1/Parkin-dependent mitophagy might be responsible for hepatic FA accumulation and treatments aimed at increasing PINK1/Parkin-mediated mitophagy (as quercetin), accelerated mitochondrial FA oxidation, and suppressed FA accumulation. Quercetin also activated PINK1/Parkin-dependent mitophagy and prevented oleic acid/palmitic acid-induced lipid accumulation in HepG2 cells (Liu et al., 2018). Linseed oil, exenatide, melatonin, akebia saponin D, and sirtuin 3 have also evidenced to reduce hepatic lipid accumulation by enhancing mitophagy (Gong et al., 2018; Li R. et al., 2018; Shao et al., 2018; Zhou et al., 2018; Yu et al., 2019). On the other hand, it also has been described that in advanced NAFLD, mitophagy increases, and mitochondrial mass, mtDNA, and PGC-1α expression are reduced; this combines with compromised ATP production, resulting in a vicious cycle of mitochondrial depletion and liver dysfunction. This later finding could mean that pharmacological interference with mitophagy molecular mechanisms may lead to therapeutic approaches (Lee et al., 2018). As can be seen, there are still serious controversies about the role of mitophagy in NAFLD that must be resolved in order to design efficient strategies in the treatment of metabolic liver diseases.

### Future Directions From the Role of Hepatic Autophagy in Metabolic Liver Diseases

Currently, existing knowledge about the autophagic response in the liver when obesity, MetS, insulin resistance, NAFLD, or diabetes are present, does not allow one to establish if a defect in the capacity of the liver to induce autophagy is the underlying cause for the appearance of such metabolic diseases. Regulation of hepatic autophagy seems to change throughout a metabolic disease, since while in the first weeks of obesity there is an increase in autophagy markers, after a few months autophagy decreases considerably (Adkins et al., 2013). In addition, although most studies suggest that a defect in liver autophagy invariably accompanies obesity, MetS, insulin resistance, and NAFLD, some reports indicate that autophagy could be overactive in diabetes (Yasuda-Yamahara et al., 2015; Choi et al., 2018; Menikdiwela et al., 2020). The need to investigate in more detail the role of autophagy in livers affected by obesity, MetS, insulin resistance, NAFLD, and/or diabetes is evident since this step is crucial for the design of new therapies that can improve the postoperative results of this type of livers when they undergo surgery and are susceptible to being damaged by I/R injury. To achieve this, first, it is important to characterize whether one or more metabolic comorbidities are present in experimental models used at present, seeing that many studies use HFD models to induce and study obesity but do not include results that allow knowing if animal model also exhibits MetS or insulin resistance (Gracia-Sancho et al., 2013; Zaouali et al., 2017; Panisello-Roselló et al., 2018). On the other hand, although there have been multiple versions of consensus within the autophagy community on the use and interpretation of assays for monitoring autophagy (Klionsky et al., 2021), it is notorious that incompliance of investigations to the established guidelines led to a discrepancy in experimental results in hepatic autophagy in the setting of metabolic diseases. For example, while various publications indicate hepatic autophagy enhancement based on results showing high levels of LC3II or Beclin-1, other investigations interpret that the reduction in levels of these parameters means an increase in autophagic activity (Parafati et al., 2015; Gong et al., 2016; Shao et al., 2018; Cui et al., 2020). Therefore, to explain controversies of existing results on hepatic autophagy in obesity, MetS, insulin resistance, NAFLD, or diabetes, it is necessary to adhere to the existing consensus on autophagy parameters, which would permit to have the most appropriate autophagy mediators in the liver to indicate with greater certainty an increase in the formation of autophagosomes and if lysosomal degradation is being carried out properly. Once the role of hepatic autophagy in these pathological conditions is more precisely understood, it will be possible to design therapeutic strategies for livers affected by metabolic diseases and undergoing surgery.

There are many studies regarding the role of autophagy in NAFLD without surgery. In them decreased liver autophagy seems to be a common finding in various experimental models of NAFLD. However, it remains to be clarified whether the reduction in autophagy is the cellular event that results in hepatic steatosis or occurs after the liver has accumulated a significant amount of lipids. It is also unknown whether autophagy changes in the function of different degrees of steatosis. Additionally, although several molecular mechanisms related to autophagy have been described in the steatotic liver, there are controversies about which ones precede autophagy failure and which ones are a consequence of such dysfunction.

Regarding the selective forms of autophagy (lipophagy and mitophagy) in livers affected by metabolic diseases, more research is mandatory. In the case of NAFLD, very few underlying molecular mechanisms for lipophagy and mitophagy have been described, whereby more research is needed in this regard, being of special interest those mediators that could be directly or indirectly related to inflammation, damage, or cell death in the liver. Moreover, given the central role of mitochondrial quality control on lipid degradation and suppression of hepatic lipid accumulation, the active search, and understanding of the signaling pathways involved in lipophagy and mitophagy in metabolic liver diseases, could yield new therapeutic options in the prevention and treatment of insulin resistance, MetS, NAFLD or diabetes in patients requiring liver surgery.

## Effects of Metabolic Syndrome and Related Diseases on Liver Surgery

Non-alcoholic fatty liver disease is the MetS-associated disease whose effects in liver surgery have been studied the most. Numerous studies have established the enormously damaging role of hepatic steatosis in I/R injury in both liver resection and transplantation. Surgical removal remains the only therapy for liver tumors with an elevated risk of failure in steatotic livers. To avoid blood loss during liver resection, portal inflow is temporarily occluded, leading to a warm ischemic period that may provoke relevant liver damage upon reperfusion. Steatosis not only accentuates the liver’s susceptibility toward ischemic insults but also hinders its regenerative capacity in terms of both repairing ischemic injury and counteracting for lost volume following resection. Together, these characteristics lead to an increased frequency of postoperative complications that restrain surgical possibilities in patients with fatty liver (Linecker et al., 2017). In addition, NAFLD makes liver grafts too sensitive to cold I/R inherent with transplantation, and thus, steatosis is correlated with a greater risk of graft malfunctioning, and consequently higher postoperative morbidity and mortality in the recipient. Because of so high a percentage of people with NAFLD, many potential donors are not eligible for donations (Forbes and Newsome, 2016). Due to the present epidemic of obesity, the repercussion of hepatic steatosis in the setting of liver surgery is expected to continue to rise (Tashiro et al., 2014).

Considering that steatotic livers tolerate I/R poorly, there is growing comprehension of molecular and cellular mechanisms underpinning the development of I/R injury in patients with fatty livers. These molecular mechanisms of damage are very different from those occurring in healthy livers (not affected by NAFLD) subjected to I/R. In this way, hepatocyte damage is markedly higher in steatotic livers than in non-steatotic ones and contributes to their poor tolerance to I/R (Selzner et al., 2000). Hepatocyte de-regulation has several causes, such as elevated susceptibility to ROS that affects mitochondrial processes and subsequently, ATP synthesis (Caraceni et al., 2005). Moreover, hepatocytes with fatty infiltration develop massive necrosis after I/R injury, rather than apoptosis observed in non-steatotic livers (Fernández et al., 2004). This fact might explicate why caspase inhibition, a protective strategy in non-steatotic livers, has no effects on hepatocyte injury in steatotic livers (Selzner et al., 2000). In experimental models of liver transplantation, exogenous nitric oxide (NO) administration reduce damage in non-steatotic grafts but is useless, or even harmful, in steatotic livers. The deleterious effects of exogenous NO are explained by exaggerated peroxynitrite generation caused by ROS overproduction (Carrasco-Chaumel et al., 2005). Steatotic livers also diverge from non-steatotic grafts in their response to the unfolded protein response (UPR) and ER stress, indeed the expression of inositol-requiring enzyme 1 (IRE1) and protein kinase R (PKR)-like endoplasmic reticulum kinase (PERK) is lower in the presence of steatosis (Ben Mosbah et al., 2010).

A very important aspect that must be contemplated, is that although in both, resection and transplantation, steatosis worsens postoperative outcomes, the signaling pathways underlying hepatic injury are substantially different in each surgical setting, this means, depending on warm or cold I/R occurs. Indeed, in each type of surgery there exist distinct therapeutic targets and therefore, treatments that work in one surgical situation might not function in the other. In accordance, increased adiponectin levels were observed in steatotic livers as a consequence of warm I/R associated with hepatic resection, and then, the treatment with adiponectin small interfering ribonucleic acid (RNA) protected steatotic livers against oxidative stress and hepatic injury (Massip-Salcedo et al., 2008). On the opposite, when subjected to transplantation, steatotic liver grafts exhibited downregulation of adiponectin. Adiponectin pre-treatment protected steatotic grafts activating the phosphoinositide-3 kinase (PI3K)/Akt pathway and unraveling AMPK as an upstream mediator of adiponectin’s actions in steatotic grafts (Jiménez-Castro et al., 2013). Similarly, a therapy based on modulating retinol binding protein 4 (RBP4) has proven to impair damage and liver regeneration in steatotic livers in the setting of hepatectomy under warm I/R; whereas the same therapeutical strategy was beneficial in steatotic livers undergoing transplantation (Casillas-Ramírez et al., 2011; Elias-Miró et al., 2012). In addition, warm I/R produced an increase in angiotensin II which injured steatotic livers, and pharmacologic blockers of angiotensin II action, such as angiotensin II receptor antagonists, protected steatotic livers against I/R injury through enhancement of peroxisome proliferator-activated receptor gamma (PPAR-γ) (Casillas-Ramírez et al., 2008). Interestingly, in liver transplantation, angiotensin II did not play a role in cold I/R in steatotic grafts, and therefore, treatment with angiotensin II receptor antagonists was useless in this type of grafts (Alfany-Fernandez et al., 2009). ER stress has shown to be a useful therapeutic target to reduce ischemic damage in steatotic livers submitted to resection under vascular occlusion (Ben Mosbah et al., 2010) but this did not happen in steatotic liver transplantation (Jiménez-Castro et al., 2012). Cortisol levels in the liver were elevated in steatotic livers undergoing resections (hepatectomy and warm I/R) and such elevations were attributed to a raise in cortisol production, and a decrease in cortisol clearance. Cortisol administration exacerbated tissue damage and regenerative failure, and such injurious effects were linked to high hepatic acetylcholine levels (Cornide-Petronio et al., 2017). Interestingly, although steatotic liver grafts also exhibited increased cortisol after transplantation due to the same causes that in hepatic resection (augment in cortisol generation and diminishment in cortisol clearance), in liver transplantation exogenous administration of cortisol treatment up-regulated the PI3K-protein kinase C (PKC) pathway, resulting in protection against the deleterious effects of brain death on damage and inflammatory response in steatotic liver transplantation (Jiménez-Castro et al., 2017). All these experimental results highlight a different role for several mediators in the regulation of damage in steatotic livers, depending on the type of surgery. This indicates that finding feasible and highly protective therapeutical strategies to reduce the adverse effects of NAFLD on liver surgery entails exhaustive evaluation of potential therapeutic targets and strategies based on its modulation in appropriated experimental models for each surgical setting.

The effects of obesity, insulin resistance, and MetS in liver surgery have not been separately investigated. There are some studies that, considering the experimental model used, one could assume that various metabolic diseases are present, such as NAFLD in combination with MetS, obesity, or insulin resistance; but unfortunately, they report confusing information about the precise diagnosis of such comorbidities. A great milestone for future research on I/R in the liver with metabolic comorbidities in appropriated experimental models would be to include parameters that facilitate the establishment of the presence of concurrent metabolic diseases and the effects of the tested treatments on them.

There are a few studies about the effects of hyperglycemia or diabetes in liver surgery. Hyperglycemia has been shown to worsen hepatic warm I/R injury by inducing hyperinflammatory immune responses through activation of advanced glycation end product (AGE)-receptor for AGE (RAGE) pathway in Kupffer cells (Wang Q. et al., 2020). Several factors may explain the mechanisms underlying the pathological and functional changes in liver injury-induced hyperglycemia, including insulin resistance, inflammation, and oxidative stress. Diabetic animals show the decreased hepatic activity of antioxidant enzymes such as catalase and SOD, thus increasing ROS, which can damage lipids, proteins, desoxyribonucleic acid (DNA), compromise mitochondria or ER function, thus leading to cell homeostasis failure and cell death. Considering that T2D is associated with hepatic lipid accumulation and that lipids are highly susceptible to being damaged by ROS, oxidative stress represents a major issue in liver function in these patients (Gjorgjieva et al., 2019). Pro-inflammatory mediators involved in hyperglycemic liver injury include interleukin (IL)-1 and IL-6, nuclear factor kappa B (NF-kB), MAPK, transforming growth factor (TGF), poly (ADP-ribose) polymerase (PARP), and TNF-α. Indeed, diabetic rat models have confirmed that in the liver, the induction of TNF-α results in increased NF-kB and JNK signaling, nitric oxide production, and apoptosis (Manna et al., 2010; Ingaramo et al., 2011). All these mediators and events occurring during oxidative stress and inflammation are part of the underlying signaling mechanisms in I/R, so when surgery occurs in the presence of hyperglycemia, the production of these mediators would be exacerbated and result in major liver injury. Inflammation and oxidative stress can negatively affect insulin sensitivity. In fact, ROS can inhibit insulin signaling by inducing Insulin Receptor Substrate (IRS) degradation in peripheral tissues, a cause of insulin desensitization (Archuleta et al., 2009). TNF-α has also been shown to induce insulin resistance (Alipourfard et al., 2019).

The negative effects of metabolic diseases in liver surgery have been described at the clinical level, and it has been shown that they can also affect a patient’s condition in the medium or long term after surgery. Transoperative stress hyperglycemia is a common clinical finding due to a transient decrease in insulin responsiveness that may persist from days to weeks after major surgery. Patients without established diabetes mellitus and who develop stress hyperglycemia, are at higher risk of poor outcomes, depending on the severity and duration of stress hyperglycemia (Chae et al., 2020). For example, hemi-hepatectomy results in moderate disturbances in glucose homeostasis that are of no clinical relevance. However, early exacerbation of insulin resistance results in a greater risk of developing overt diabetes in the long term (Durczynski et al., 2013). Along the same lines, diabetes negatively impacts the surgical outcome of patients with cirrhosis after liver resection and transplantation, and the stress associated with the post-reperfusion syndrome further increases hyperglycemia and insulin resistance during surgery in recipients with reduced-size liver grafts (Chae et al., 2020). Although the exact mechanisms responsible for these adverse outcomes are not completely elucidated, a probable cause seems to be the increase in I/R injury resulting from the acute hyperglycemic disturbances. As a result, the early post-reperfusion period is critical since significant hepatic insults are inflicted, and graft regeneration is concurrently initiated with metabolic and detoxifying associated phenomena (Kang et al., 2018). Moreover, liver transplantation recipients have been shown to progressively develop MetS in a high proportion despite current efforts to mitigate their evolution (i.e., lifestyle modifications and aggressive management of hypertension, diabetes, and hyperlipidemia). Associated risk factors include age, increased body mass index, and pre- and post-transplantation serum glucose (Vida Perez et al., 2016). Importantly, increased recurrence of NASH and cryptogenic cirrhosis was associated with the presence of concomitant MetS, hypertension, and the use of insulin. Recurrence should be further evaluated in larger studies, with special emphasis on the management of MetS and prevention strategies (El Atrache et al., 2012). Curtailing the development of postoperative MetS after orthotopic liver transplantation, may decrease readmissions and improve patient and graft outcomes (Chang et al., 2016).

## Autophagy in Liver Surgery in the Setting of Metabolic Syndrome

The majority of research about autophagy in liver surgery has been focused on healthy livers more than on livers affected by MetS or related metabolic diseases. Such investigations have allowed us to distinguish the relevance of autophagy in liver surgery outcomes and a better mechanistic understanding of the autophagy process in resection and transplantation, which makes it possible to conceive new therapeutic options that could be evaluated in livers suffering metabolic diseases and submitted to surgery. In some cases, findings on autophagy in I/R injury in healthy livers have been reflected in the clinical setting. This supports that results in autophagy in liver surgery in the setting of metabolic diseases might be translated from the laboratory toward a possible new clinical therapy. Therefore, this section first discusses the existing knowledge on the role of autophagy in liver surgery without the effect of MetS or related diseases; subsequently, published results regarding the role of autophagy in I/R in livers with MetS and associated diseases are analyzed; and finally, new lines of research that could be approached in the future to improve the results of liver surgery in the setting of metabolic diseases are discussed.

### Autophagy in Hepatic I/R Injury

The pathogenic signaling pathways in hepatic I/R have been documented to be different in normothermic *vs*. cold ischemia, and even in livers in optimal conditions and those with underlying pathological conditions such as steatosis. Then, it is foreseeable that the autophagy process also works differently in I/R injury in livers depending on whether they undergo resection or transplantation. The design of therapeutic strategies based on autophagy modulation to protect livers suffering metabolic disorders and subjected to surgery, largely depends on a clear understanding of the molecular mechanisms of autophagy in liver I/R injury, distinguishing between liver resection and transplantation models.

#### Autophagy in Warm I/R Injury Associated With Hepatic Resection

Many reports in the literature strongly support a relevant role of autophagy in I/R injury in liver resection. Recent research on this topic has focused on the discovery of novel molecular signaling mechanisms that, in the first instance, are being studied in experimental models with optimal livers (without steatosis or other metabolic comorbidities). Table 1 summarizes the studies in the literature on autophagy in warm I/R that have been conducted in the last 5 years. As shown, results have been contradictory, since some indicate that increased autophagy protects livers subjected to I/R, while others report that it is the inhibition of autophagic activity that leads to beneficial effects. These findings are most likely due to the different experimental models used, including the different durations of ischemia and/or reperfusion. It is widely known that experimental conditions influence the mechanisms underlying liver I/R, and hence, the results.

**TABLE 1:** Outcomes about the role of autophagy in normothermic hepatic I/R injury, in the last 5 years.

Study	Animal species	Type of liver	Ischemia time	Reperfusion time	Parameters of autophagy in normothermic ischemia without modulation	Modulation of autophagy	Results from autophagy modulation vs. untreated groups
Lee et al., 2016	Hepatocytes from AML12 cell line	Optimal	1 h	0, 1, 3, 5, and 24 h	Autophagy parameters vs. Control group: ↓LC3 I to LC3II conversion, ↓Atg5. ↑mTOR phosphorylated.	Yes. Everolimus.	Autophagy enhancement: ↑LC3 I to LC3 II conversion, ↓p62. Cell injury: ↓Apoptosis.
Biel et al., 2016	C57BL/6 mice primary hepatocytes	Optimal	4 h	1 and 2 h	Autophagic parameters vs. Control group: Mild ↑LC3 I to LC3 II conversion. No expression of SIRT1	Yes. SIRT1 overexpression	Autophagy enhancement: ↑LC3 I to LC3 II conversion, ↑Autophagosomes number. ↑Mitophagy: Improved mitochondria structure and function. Cell injury: ↓Cell death percentage.
Xu D. et al., 2017	C57BL/6 mice primary hepatocytes	Optimal	4 h	2 h	Autophagy parameters vs. Control group: Mild ↑LC3 I to LC3 II conversion, mild ↑SQSTM1. ↑Autophagosomes number.	Yes. CDDO imidazole, a Nrf2 activator.	Autophagy enhancement: ↑LC3 I to LC3 II conversion, ↑Autophagosomes number, ↓SQSTM1. ↓Mitochondrial dysfunction. Cell injury: ↓Cytotoxicity percentage, ↓Apoptosis.
Khader et al., 2017	H4IIE hepatoma cells and Sprague-Dawley rat primary hepatocytes	Optimal	4 and 6 h	2, 4, and 24 h	Autophagy parameters vs. Control group: ↓LC3 I to LC3 II conversion.	Yes. SRT1720, a SIRT1 activator.	Autophagy enhancement: ↑LC3 I to LC3 II conversion, ↓SQSTM1. ↓Mitochondrial dysfunction. Cell injury: ↑Cell survival.
Cui et al., 2018	Hepatocytes from AML12 cell line	Optimal	90 min	12 h	Autophagy parameters: Notable LC3 and Beclin-1 expression.	Yes. Interferon regulatory factor-1 siRNA or Glycyrrhizin Acid, an HMGB1 inhibitor.	Autophagy inhibition: ↓LC3 and ↓Beclin-1 expression.
Kong et al., 2019	C57BL/6 mice primary hepatocytes	Optimal	4 h	2 h	Autophagy parameters: Mild LC3 I to LC3 II conversion and ATG5 expression. Notable expression of mTOR phosphorylated.	Yes. SB216763, inhibitor of GSK3β.	Autophagy enhancement: ↑LC3 I to LC3 II conversion, ↑ATG5. ↓mTOR phosphorylated. Cell injury: ↓Cytotoxicity percentage.
Li J. et al., 2020	C57BL/6 mice primary hepatocytes	Optimal	Not specified.	Not specified.	Autophagy parameters vs. Sham group: ↓ LC3 I to LC3 II conversion, ↓ATG7.	Yes. CD5-like (CD5L) protein, an apoptosis inhibitor of macrophage.	Autophagy enhancement: ↑LC3 I to LC3 II conversion, ↑ATG7. Cell injury: ↓Apoptosis, ↓Oxidative stress.
Bi et al., 2020	Sprague-Dawley rat primary hepatocytes	Aged hepatocytes	1 h	8 h	Autophagy parameters vs. Control old group: Mild ↑LC3 I to LC3 II conversion and mild ↓p62.	Yes. Irisin.	Autophagy enhancement: ↑LC3 I to LC3 II conversion, ↓p62.
Lee et al., 2016	BALB/c mice.	Optimal	Partial normothermic ischemia (right lateral lobe). 45 min	24 h	Autophagy parameters vs. Sham group: ↓LC3B, mild ↑p62.	Yes. Everolimus.	Autophagy enhancement: ↑ LC3B, ↓p62. Liver damage: ↓ALT and AST, ↓Necrosis, ↓Apoptosis, ↓Inflammation.
Li M. et al., 2018	C57BL/6 mice	Optimal	Partial normothermic ischemia (right lateral lobe). 90 min	6 and 12 h	Autophagy parameters vs. Sham group: No changes in LC3 I to LC3II conversion or autophagosomes number, mild ↑p62.	Yes. Alda-1, an activator of ALDH2.	Autophagy enhancement: ↑LC3 I to LC3 II conversion, ↑Autophagosomes number, ↓p62. Liver damage: ↓ALT and AST, ↓Necrosis, ↓Apoptosis, ↓Inflammation, ↓Oxidative stress.
Xiang et al., 2020	Balb/c mice	Optimal	Partial normothermic ischemia of 70% (left and middle Lobes). 1 h	2, 8, and 24 h	Autophagy parameters vs. Sham group: ↑Beclin-1, ↑LC3-II, ↓p62.	Yes. Bergenin.	Autophagy inhibition: ↓Beclin-1, ↓LC3-II, ↑p62. Liver damage: ↓ALT and AST, ↓Necrosis, ↓Apoptosis, ↓Inflammation, ↓Oxidative stress.
Ji et al., 2020	BALB/c mice	Optimal	Partial normothermic ischemia of 70% (left and middle lobes). 45 min	2, 8, and 24 h	Autophagy parameters vs. Sham group: ↑Beclin-1, ↑LC3, ↓p62.	Yes. Cafestol, a natural diterpene extract from coffee beans.	Autophagy inhibition: ↓Beclin-1, ↓LC3, ↑p62. Liver damage: ↓ALT and AST, ↓Necrosis, ↓Apoptosis, ↓Inflammation.
Tan et al., 2018	C57BL/6 mice	Optimal	Partial normothermic ischemia of 70% (left and middle lobes). 1 h	6 h	Autophagy parameters: Mild LC3 I to LC3 II conversion and Beclin-1 expression. Low number of autophagosomes. Notable expression of phosphorylated mTOR.	Yes. Helix B surface peptide, an erythropoietin -derived peptide.	Autophagy enhancement: ↑LC3 I to LC3 II conversion, ↑Beclin-1, ↑Autophagosomes number. Liver damage: ↓ALT and AST, ↓Necrosis, ↓Apoptosis.
Li H. et al., 2020	Sprague-Dawley rats	Optimal	Partial normothermic ischemia of 70% (left and middle lobes). 45 min	6 h	Autophagy parameters vs. Sham group: ↑LC3, ↑Beclin-1, ↑ATG-7, ↓p62. Affected mitochondrial structure.	Yes. N-acetyl-L-tryptophan, a ROS scavenger.	Autophagy inhibition: ↓LC3, ↓Beclin-1, ↓ ATG-7, ↑p62. ↓Mitophagy: Improved mitochondria morphology.
Kong et al., 2019	C57BL/6 mice	Optimal	Partial normothermic ischemia (cephalad lobes). 90 min	6 h	Autophagy parameters vs. Sham group: ↑LC3 I to LC3 II conversion, ↑ATG5, ↑Autophagosomes number. ↑mTOR phosphorylated.	Yes. SB216763, an inhibitor of GSK3β.	Autophagy enhancement: ↑LC3 I to LC3 II conversion, ↑ATG5, ↑Autophagosomes number. ↓ mTOR phosphorylated. Liver damage: ↓ALT and AST, ↓Necrosis, ↓Apoptosis.
Wu et al., 2017	Balb/c mice	Optimal	Partial normothermic ischemia of 70% (left and middle lobes). 45 min	2, 8, and 24 h	Autophagy parameters vs. Sham group: ↑Beclin-1, ↑LC3, ↓p62.	Yes. Quercetin.	Autophagy inhibition: ↓Beclin-1, ↓LC3, ↑p62. Liver damage: ↓ALT and AST, ↓Necrosis, ↓Apoptosis, ↓Inflammation.
Biel et al., 2016	C57BL/6 mice	Optimal	Total hepatic ischemia. 45 min	20 min	Autophagy parameters vs. Control group: ↓LC3 I to LC3 II conversion. ↓SIRT1	Yes. SIRT1 overexpression	Autophagy enhancement: ↑LC3 I to LC3 II conversion. ↑Mitophagy: Improved mitochondria function.
Liu et al., 2020	Sprague-Dawley rats	Optimal	Partial normothermic ischemia of 70% (left and middle lobes). 1 h	6 and 24 h	Autophagy parameters vs. Sham group: ↓LC3 I to LC3 II conversion, ↓ ATG7, ↑p62. ↑mTOR phosphorylated.	Yes. Alda-1, an activator of ALDH2.	Autophagy enhancement: ↑LC3 I to LC3 II conversion, ↑ATG7, ↓p62. ↓mTOR phosphorylated Liver damage: ↓ALT and AST, ↓Necrosis, ↓Apoptosis, ↓Inflammation, ↓Oxidative stress.
Yang et al., 2015	C57BL/6 mice	Optimal	Partial normothermic ischemia of 70% (left and middle lobes). 1 h	6 h	Autophagy parameters vs. Sham group: Mild ↑LC3I to LC3II conversion, mild ↑Beclin-1, mild ↑ATG7 and mild ↑Autophagic vacuoles.	Yes. Vitamin D	Autophagy enhancement: ↑LC3 I to LC3 II conversion, ↑Beclin-1, ↑ATG7, ↑autophagic vacuoles. Liver damage: ↓ALT and AST, ↓Necrosis, ↓Apoptosis, ↓Inflammation, ↓Oxidative stress.
Chen et al., 2017a	Balb/c mice	Optimal	Partial normothermic ischemia of 70% (left and middle lobes). 1 h	6, 12, and 24 h	Autophagy parameters vs. Sham group: ↑LC3I to LC3II conversion, ↑Beclin-1.	Yes. 15-Deoxy-Δ12,14-prostaglandin J2, a dehydration product of prostaglandin D2.	Autophagy inhibition: ↓LC3 I to LC3 II conversion, ↓Beclin-1. Liver damage: ↓ALT and AST, ↓Necrosis, ↓Apoptosis, ↓Inflammation, ↓Oxidative stress.
Feng et al., 2017	Balb/c mice	Optimal	Type of normothermic ischemia not specified. 45 min	2, 8, and 24 h	Autophagy parameters vs. Sham group: ↑LC3 I to LC3 II conversion, ↑Beclin-1, ↓p62, ↑Autophagosomes formation. ↓mTOR phosphorylated	Yes. Salidroside, main active component of *Rhodiola rosea*.	Autophagy inhibition: ↓LC3 I to LC3 II conversion, ↓Beclin-1, ↑p62, ↓Autophagosomes formation. ↑mTOR phosphorylated Liver damage: ↓ALT and AST, ↓Necrosis, ↓Apoptosis, ↓Inflammation.
Deng et al., 2018	Balb/c mice	Optimal	Partial normothermic ischemia of 70% (ischemic lobes not specified). 45 min	2, 8, and 24 h	Autophagy parameters vs. Sham group: ↑LC3 I to LC3 II conversion, ↑Beclin-1, ↓p62.	Yes. Beraprost sodium, an analog of prostacyclin.	Autophagy inhibition: ↓LC3 I to LC3 II conversion, ↓Beclin-1, ↑p62. Liver damage: ↓ALT and AST, ↓Necrosis, ↓Apoptosis, ↓Inflammation.
Cui et al., 2018	C57BL/6 mice	Optimal	Partial normothermic ischemia of 70% (left and middle lobes). 90 min	2, 6, 12, and 24 h	Autophagy parameters vs. Sham group: ↑LC3 I to LC3 II conversion, ↑Autophagosomes number.	Yes. Knockout of Interferon regulatory factor-1.	Autophagy inhibition: ↓LC3 I to LC3 II conversion, ↓Autophagosomes number. Liver damage: ↓ALT and AST, ↓Necrosis.
Kou et al., 2020	Sprague Dawley rats	Optimal	Partial normothermic ischemia of 70% (left and middle lobes). 90 min	6 h	Autophagy parameters vs. Sham group: ↑LC3 I to LC3 II conversion, ↑Beclin-1.	Yes. Glycyrrhizin, an HMGB1 inhibitor.	Autophagy inhibition: ↓LC3 I to LC3 II conversion, ↓Beclin-1. Liver damage: ↓ALT and AST, ↓Necrosis, ↓Apoptosis, ↑NO, ↓Endothelin-1, ↓Inflammation, ↓Oxidative stress.
Yu et al., 2019	BALB/c mice	Optimal	Partial normothermic ischemia of 70% (ischemic lobes not specified). 45 min	2, 8, and 24 h	Autophagy parameters vs. Sham group: ↑LC3 and ↑Beclin-1.	Yes. Levo-tetrahydropalmatine (L-THP), an active component of *Corydalis yanhusuo*.	Autophagy inhibition: ↓LC3, ↓Beclin-1. Liver damage: ↓ALT and AST, ↓Necrosis, ↓Apoptosis, ↓Inflammation.
Dusabimana et al., 2019)	C57BL/6 mice	Optimal	Partial normothermic ischemia of 70% (left and middle lobes). 1 h	5 h	Autophagy parameters vs. Sham group: No changes in LC3 I to LC3 II conversion or ATG5. ↓ATG12, ↑p62. ↓SIRT1/FOXO3a	Yes. Nobiletin, a natural flavonoid.	Autophagy enhancement: ↑LC3 I to LC3 II conversion, ↑ATG5, ↑ATG12, ↓p62. ↑SIRT1/FOXO3a Liver damage: ↓ALT and AST, ↓Necrosis, ↓Apoptosis, ↓Inflammation, ↓Oxidative stress.
Xu D. et al., 2017	C57BL/6 mice	Optimal	Partial normothermic ischemia of 70% (cephalad lobes). 90 min	6 and 12 h	Autophagy parameters vs. Sham group: Mild ↑LC3 I to LC3 II conversion, ↑Autophagosomes number, ↑SQSTM1.	Yes. CDDO imidazole, a Nrf2 activator.	Autophagy enhancement: ↑LC3 I to LC3 II conversion, ↑Autophagosomes number, ↓SQSTM1. Liver damage: ↓ALT and AST, ↓Necrosis, ↓Apoptosis, ↓Inflammation, ↓Oxidative stress.
Wang et al., 2019	BALB/c mice	Optimal	Partial normothermic ischemia of 70% (left and middle lobes). 1 h	2, 8, and 24 h	Autophagy parameters vs. Sham group: ↑LC3 I to LC3 II conversion, ↑Beclin-1.	Yes. Oleanolic Acid.	Autophagy inhibition: ↓LC3 I to LC3 II conversion, ↓Beclin-1. Liver damage: ↓ALT and AST, ↓Necrosis, ↓Apoptosis.
Xu S. et al., 2017	Balb/c mice	Optimal	Partial normothermic ischemia of 70% (ischemic lobes not specified). 45 min	2, 8, and 24 h	Autophagy parameters vs. Sham group: ↑LC3 II, ↑Beclin-1, ↓p62.	Yes. Propylene glycol alginate sodium sulfate, a polysaccharide isolated from brown algae.	Autophagy inhibition: ↓LC3 II, ↓Beclin-1, ↑p62. Liver damage: ↓ALT and AST, ↓Necrosis, ↓Apoptosis, ↓Inflammation.
Liu H. et al., 2019	C57BL/6 mice	Optimal	Partial normothermic ischemia of 70% (left and middle lobes). 1 h	8, 12, and 24 h	Autophagy parameters vs. Sham group: Mild ↑LC3 I to LC3 II conversion, mild ↑Beclin-1 and mild ↑Autophagic vacuoles number. Mild ↑AMPK phosphorylated, Mild ↑ULK-1 phosphorylated. ↑mTOR phosphorylated.	Yes. Spermidine	Autophagy enhancement: ↑LC3 I to LC3 II conversion, ↑Beclin-1, ↑Autophagic vacuoles number. ↑ULK-1 phosphorylated, ↓mTOR phosphorylated. Liver damage: ↓ALT and AST, ↓Necrosis, ↓Apoptosis, ↓Inflammation.
Li J. et al., 2017	BALB/C mice	Optimal	Partial normothermic ischemia of 70% (left and middle lobes). 1 h	2, 8, and 24 h	Autophagy parameters vs. Sham group: ↑LC3 I to LC3 II conversion, ↑Beclin-1, ↓p62, ↑Autophagosomes and Autolysosomes number.	Yes. Fucoidan.	Autophagy inhibition: ↓LC3 I to LC3 II conversion, ↓Beclin-1, ↑p62, ↓Autophagosomes and Autolysosomes number. Liver damage: ↓ALT and AST, ↓Necrosis, ↓Apoptosis, ↓Inflammation.
Miyauchi et al., 2019	C57BL/6 mice	Optimal	Partial normothermic ischemia of 70% (left and middle lobes). 1 h	1, 3, and 6 h	Autophagy parameters: Mild LC3B expression. Mild FOXO1/3 expression.	Yes. 12-h fasting β-hydroxybutyric acid	Autophagy enhancement: ↑LC3B ↑FOXO1/3 Liver damage: ↓ALT, ↓Necrosis, ↓Apoptosis, ↓Inflammation, ↓Oxidative stress.
Bai et al., 2018	Bama miniature pigs.	Optimal	Partial normothermic ischemia combined with partial resection. Ischemia time: 1 h	3 h, 1 and 3 days	Autophagy parameters vs. Sham group: ↑LC3B, ↑Beclin-1 ↓p62. ↓mTOR	Yes. Hydrogen-rich saline.	Autophagy inhibition: ↓LC3B, ↓Beclin-1, ↑p62. ↑mTOR Liver damage: ↓ALT and AST, ↓Oxidative stress.
Khader et al., 2017	C57BL/6 mice	Optimal	Partial normothermic ischemia of 70% (left and middle lobes). 1 h	24 h	Autophagy parameters vs. Control group: No changes in LC3 I to LC3 II conversion or SQSTM1.	Yes. SRT1720, a SIRT1 activator.	Autophagy enhancement: ↑LC3 I to LC3 II conversion, ↓SQSTM1. Liver damage: ↓ALT and AST, ↓Necrosis, ↓Apoptosis, ↓Inflammation, ↓Oxidative stress.
Jiang et al., 2019	C57BL/6 mice	Aged livers	Partial normothermic ischemia of 70% (cephalad lobes). 90 min	6 h	Autophagy parameters vs. Sham group: ↓ LC3 I to LC3 II conversion and ↑p62.	Yes. Ischemic preconditioning combined with Rapamycin.	Autophagy enhancement: ↑LC3 I to LC3 II conversion, ↓p62. Liver damage: ↓ALT, ↓Necrosis, ↓Apoptosis
Bi et al., 2020	Sprague-Dawley rats	Aged liver	Partial normothermic ischemia of 70% (ischemic lobes not specified). 60 min	24 h	Autophagy parameters vs. Sham old group: ↑LC3 I to LC3 II conversion and ↓p62.	Yes. Irisin.	Autophagy enhancement: ↑LC3 I to LC3 II conversion, ↓p62. Liver damage: ↓ALT, ↓Necrosis, ↓Apoptosis, ↓Inflammation, ↓Oxidative stress.
Cho et al., 2017	C57BL/6 mice	Alcoholic fatty liver	Partial normothermic ischemia of 70% (left and middle lobes). 1 h	5 h	Autophagy parameters vs. Sham group: ↓LC3 I to LC3 II conversion, ↓ATG3, ↓ATG7, ↓ATG12-5, ↑p62, ↓LAMP-2, ↓Autophagic vacuoles number. ↓Mitophagy: ↑PINK1 and ↓Parkin. ↓SIRT1	Yes. 2-Methoxyestradiol	Autophagy enhancement: ↑LC3 I to LC3 II conversion, ↑ATG3, ↑ATG7, ↑ATG12-5, ↓p62, ↑LAMP-2 ↑Autophagic vacuoles number. ↑Mitophagy: ↓PINK1 and ↑Parkin ↑SIRT1 Liver damage: ↓ALT and AST, ↓Necrosis, ↓Inflammation,

Some findings in signaling molecular pathways lately described in warm ischemia are included following. Long-non-coding RNAs (lncRNAs) homeobox (HOX) transcript antisense RNA (HOTAIR), regulated autophagy via the microRNA (miR)-20b-5p/ATG7 axis in I/R injury in mice subjected to partial hepatic ischemia (Tang et al., 2019). HOTAIR and ATG7 expression levels increased as did autophagy during I/R injury, while the knockdown of HOTAIR expression attenuated autophagy in isolated hepatocytes (Tang et al., 2019). During experimental hepatic I/R injury, the master transcription factor Interferon Regulatory Factor (IRF1) was upregulated and associated with activation of autophagic signaling (Yu et al., 2017). IRF1 also inhibited β-catenin expression in livers subjected to I/R injury, it activated autophagy and worsened hepatic injury (Yan et al., 2020). MicroRNA refers to a highly conserved, small, non-coded RNA, associated with basic cellular processes, such as apoptosis, proliferation, and stress responses. MicroRNAs have been shown to also play a role in autophagy regulation. *In vivo* and *in vitro* miR-30b levels are down-regulated after hepatic I/R injury, and simultaneously, activate autophagy. High levels of miR-17 upregulate autophagy and worsen hepatic I/R injury by suppressing Stat3 expression *in vitro* (Li et al., 2016). Interestingly, mitophagy is involved in warm hepatic ischemia, and some molecular mediators related to this process are the following: (a) Parkin expression and mitochondrial autophagy are up-regulated after I/R injury (Ning et al., 2018); (b) PINK1 (a mediator of mitophagy) protected against hepatic I/R injury by preventing nucleotide-binding oligomerization domain (NOD)-like receptor (NLR) family pyrin domain containing 3 (NLRP3) inflammasome activation in mice subjected to partial warm I/R (Xu et al., 2020), and (c) miR-330-3p suppresses phosphoglycerate mutase family member 5 (PGAM5)-induced mitophagy to dampen hepatic I/R injury (Sun et al., 2019).

#### Autophagy in Cold I/R Injury Inherent to Liver Transplantation

Table 2 summarizes the studies evaluating the role of autophagy in liver transplantation. In optimal liver transplantation models subjected to various cold storage times, a slight increase or no change in autophagy parameters are usually observed as a result of ischemic insult. The various strategies that have been used to increase autophagy activity have proven to be beneficial in decreasing liver injury and cell death (Pantazi et al., 2015; Nakamura et al., 2018, 2019; Wang J. et al., 2020). Only one study revealed that pharmacological inhibition of autophagy protected optimal grafts undergoing transplantation (Gotoh et al., 2009). However, it should be noted that in this study, the autophagy and liver injury parameters were evaluated after very short reperfusion periods (15 or 120 min) in comparison with the rest of the studies (6 to 24 h). In studies using allogeneic optimal liver grafts (Lewis rat donor and Norway rat recipient), there are disagreements. In a study based on transplantation with a small-size liver graft, a discrete increase in some autophagy parameters was detected, and the grafts were protected by increasing autophagic activity through cell therapy combined with gene overexpression; this led to a decrease in the rejection rate. In another study, benefits for graft viability (reduction of injury and graft rejection) were obtained by administering drugs to inhibit autophagy (Wang R.R. et al., 2017; Chen et al., 2019b). In these cases, the main difference that could explain the contradictory results was the decrease in the liver graft size in one of the models. Small grafts are known to exhibit a much greater hepatic regeneration response than whole-size grafts (González et al., 2010). Perhaps, mechanisms underlying regeneration may influence other signaling pathways including autophagy; therefore, autophagy modulation in small-size transplants might induce different effects on liver injury than in whole-size transplants.

**TABLE 2:** Findings about the involvement of autophagy in liver transplantation.

Study	Animal species	Type of liver graft	Cold preservation time	Reperfusion time	Parameters of autophagy in transplantation without modulation	Modulation of autophagy	Results from autophagy modulation vs. untreated groups
Pantazi et al., 2015	Male SD rats	Optimal grafts	8 h	24 h	Autophagy parameters vs. Sham group: No changes in LC3B or Beclin-1. Mild ↑SIRT1/FOXO1 pathway activity. No changes in mTOR activity.	YES. Trimetazidine added to IGL-1 preservation solution.	Autophagy enhancement: ↑ LC3B and ↑Beclin-1. ↑SIRT1/FOXO1 pathway activity, ↓mTOR activity. Liver damage: ↓ALT, ↓Oxidative stress.
Nakamura et al., 2019	C57BL/6 mice	Optimal grafts in mice	18 h in mice	6 h	Autophagy parameters: Mild LC3 I to LC3II conversion. ↑ p-S6K (mTORC1 activity) and ↑CHOP (ER stress marker).	YES. Antibiotic pretreatment in recipient to modulate gut microbiome.	Autophagy enhancement: ↑LC3 I to LC3 II conversion. ↓ p-S6K (mTORC1 activity) and ↓ER stress (CHOP). Liver damage: ↓AST, ↓Necrosis, ↓Apoptosis, ↓Inflammation.
Xue et al., 2020	C57BL/6 mice	Optimal graft	20 h	6 h	Autophagy parameters vs. Sham group: ↓LC3 I to LC3II conversion, ↑ATG5 ↑Beclin-1, no changes in p62.	YES. Pituitary adenylate cyclase-activating polypeptide (PACAP).	Autophagy enhancement: ↑LC3 I to LC3 II conversion, ↑ATG5, ↑Beclin-1, ↓p62. Liver damage: ↑ Survival rate, ↓ALT, ↓Necrosis.
Nakamura et al., 2018	C57BL/6 mice	Optimal grafts in mice	20 h	6 h	Autophagy markers: Mild expression of LC3B and SIRT1.	YES. Heme oxygenase-1 overexpression.	Autophagy enhancement: ↑LC3B. ↑SIRT1. Liver damage: ↓ALT, ↓Necrosis, ↓Apoptosis, ↓Inflammatory mediators.
Gotoh et al., 2009	Wistar rats	Optimal grafts	24 h	15 and 120 min	Autophagy markers: Detected LC3 and nascent autophagosomes and autolysosomes, at 15 min of reperfusion.	YES. Wortmannin, a PI3K inhibitor.	Autophagy inhibition: ↓LC3, ↓Number of nascent autophagosomes and autolysosomes volume density, at 15 min of reperfusion. Liver damage: ↓ALT and AST at 120 min of reperfusion, ↑ Survival rate.
Wang J. et al., 2020	SD rats	Optimal graft	Not specified.	24 h	Autophagy parameters vs. Sham group: No changes in LC3 I to LC3II conversion, ATG5 or ATG16L1. ↑p62. ↑AKT/mTOR	YES. Suberoylanilide hydroxamic acid (SAHA), a pan-histone deacetylase inhibitor.	Autophagy enhancement: ↑LC3 I to LC3 II conversion, ↑ATG5, ↑ATG16L1, ↓p62. ↓AKT/mTOR Liver damage: ↓ALT and AST, ↓Apoptosis, ↓Inflammatory cytokines.
Lin et al., 2017	Wistar rats	Optimal grafts	Not specified	6 h	Autophagy parameters vs. Sham group: Mild ↑LC3 I to LC3 II conversion, ↑Beclin-1, mild ↓p62. Mild ↑SIRT1/FoxO3α pathway activity.	YES. Berberine.	Autophagy enhancement: ↑LC3 I to LC3 II conversion, ↑Beclin-1, ↓p62. ↑SIRT1/FoxO3α pathway activity. Liver damage: ↓ALT and AST, ↓Apoptosis, ↓Oxidative stress.
Chen et al., 2019b	Lewis rats as donors and Brown Norway rats as recipients.	Optimal grafts	35 min	14 days	Autophagy parameters vs. syngeneic control: ↑LC3 I to LC3 II conversion in CD8 + T cells.	YES. 3-Methyladenine, an autophagy inhibitor.	Autophagy inhibition: ↓LC3 I to LC3 II conversion in CD8 + T cells. Liver damage: ↓ALT and AST, ↓Rejection index, ↑ Survival rate.
Wang R. R. et al., 2017	Lewis rats as donors and Brown Norway rats as recipients.	Reduced-size liver grafts.	Not specified	0, 1, 3, 5, 7, and 14 days	Autophagy parameters: Mild LC3 I to LC3 II conversion and Beclin-1 expression. Notable expression of phosphorylated mTOR.	YES. HO-1 transduced Bone-marrow derived Mesenchymal Stem Cells.	Autophagy enhancement: ↑LC3 I to LC3 II conversion, ↑Beclin-1. ↓mTOR phosphorylated. Liver damage: ↓Rejection index, ↓Apoptosis.
Zeng et al., 2019	C57BL/6 mice	Grafts from donation after circulatory death (DCD).	4 h	2 h	Autophagy parameters vs. control: Mild ↑LC3B-II ULK1, mild ↑Atg5 and mild ↓p62. ↓mTOR. Not statistical significance.	YES. HOPE (Hypothermic oxygenated machine perfusion) treatment during 1 h	Autophagy enhancement: ↑LC3B-II,↑ULK1, ↑Atg5 and ↓p62. ↓mTOR. Liver damage: ↓ALT and AST, ↓ Necrosis and Apoptosis, ↓Oxidative stress.
Liu T. et al., 2019	Sprague Dawley rats.	Grafts from DCD.	Not specified	6, 12, and 24 h	Autophagy parameters at 6, 12, and 24 h of reperfusion in rats: Notable increased expression of LC3 and AMPK. Notable decreased expression of p62.	NO.	N/A
Zhang et al., 2019	Wistar rats fed with high-fat diet for 12 weeks.	Steatotic grafts. Not additional data about presence of metabolic comorbidities.	Not specified	6 h	Autophagy parameters vs. steatotic sham: ↑LC3 I to LC3 II conversion, ↑Beclin-1, ↑p62, ↑Autophagosomes number. ↑ER stress (p-PERK, CHOP, Bip).	YES. Berberine.	Autophagy inhibition: ↓LC3 I to LC3 II conversion, ↓Beclin-1, ↓p62, ↓Autophagosomes number. ↓ER stress (p-PERK, CHOP, Bip). Liver damage: ↓ALT and AST, ↓Necrosis, ↓Oxidative stress, ↓Inflammatory cytokines.
Panisello-Roselló et al., 2018	Zucker rats	Steatotic grafts. Obesity.	24 h	Not reperfusion. Liver samples collected at the end of cold storage.	Autophagy parameters vs. steatotic sham: No changes in, LC3B, Beclin-1 or ATG7. ↓mTOR activity	YES. IGL-1 preservation solution.	Autophagy enhancement: ↑ LC3B,↑Beclin-1, ↑ATG7. ↓mTOR activity. Liver damage: ↓ALT and AST, ↓Necrosis, ↓Apoptosis.
Gracia-Sancho et al., 2013	Wistar rats fed with high-fat diet for 3 days.	Steatotic grafts. Not additional data about presence of metabolic comorbidities.	16 h	1 h (*ex vivo* reperfusion)	Autophagy parameters vs. steatotic control: Mild ↓LC3 I to LC3 II conversion.	YES. Simvastatin.	Autophagy enhancement: ↑LC3 I to LC3 II conversion. Liver damage: ↓ALT and AST.
Zaouali et al., 2017	Zucker rats	Steatotic grafts. Obesity.	24 h	2 h (*ex vivo* reperfusion)	Autophagy parameters vs. steatotic control: No changes in LC3 I to LC3 II conversion, Beclin-1 No changes in SIRT1.	YES. Trimetazidene added to IGL-1 preservation solution.	Autophagy enhancement: ↑LC3 I to LC3 II conversion, ↑Beclin-1. ↑SIRT1. Liver damage: ↓ALT and AST ↓TNFα.
Zaouali et al., 2013b	Zucker rats	Steatotic grafts. Obesity.	24 h	2 h (*ex vivo* reperfusion)	Autophagy parameters vs. steatotic control: Mild ↑LC3 I to LC3 II conversion, ↑Beclin-1, ↑p62. ↑ER stress (GRP78, CHOP, p-PERK).	YES. Trimetazidene + Melatonin added to IGL-1 preservation solution.	Autophagy enhancement: ↑LC3 I to LC3 II conversion, ↑Beclin-1, ↑ATG7, ↓p62. ↓ER stress (GRP78, CHOP, p-PERK). Liver damage: ↓ALT and AST, ↓Oxidative stress.
Minor et al., 2011	German landrace pigs	Optimal grafts.	10 h	1 h, 7 days	Autophagy parameters: Mild LC3 II and Beclin-1 expression, at 1 h of reperfusion.	YES. Hypothermic reconditioning by gaseous oxygen persufflation treatment during 2 h.	Autophagy enhancement: ↑LC3-II, ↑Beclin-1, at 1 h of reperfusion. Liver damage: ↓ALT ↑ Survival rate, at 7 days after transplantation.
Liu T. et al., 2019	Bama miniature pigs	Grafts from DCD.	Not specified	6, 12, and 24 h	Autophagy parameters at 24 h of reperfusion in pigs: Notable increased expression of LC3 and AMPK. Notable decreased expression of p62.	NO.	N/A
Nakamura et al., 2019	Humans	Grafts from donors after brain or cardiac death.	7–8 h on average	2 h	Autophagy parameters: Mild LC3B expression. Notable CHOP expression.	YES. Pre-transplantation Antibiotics treatment ≥ 10 days in recipients.	Autophagy enhancement: ↑LC3B. ↓ER stress (CHOP). Liver damage: ↓ALT and AST.
Chen et al., 2019b	Humans	Liver transplant recipients with acute rejection.	Not specified	Not specified	Autophagy parameters vs. recipients without acute rejection: ↑LC3 expression in CD8 + T cells.	NO	N/A
Nakamura et al., 2018	Humans	Not specified	Not specified	2 h after portal reperfusion	Autophagy markers: Expression of LC3B and SIRT1.	NO	N/A
Ricca et al., 2015	Humans	Grafts from donors after brain death, including steatotic and non-steatotic liver grafts.	5 to 10 h	≤2 h after reperfusion	Autophagy markers: Expression of LC3.	YES. Ischemic postconditioning.	Autophagy enhancement: ↑LC3. Liver damage: ↓I/R injury at reperfusion biopsy, defined by the presence of both inflammatory infiltration and hepatocellular necrosis.
Degli Esposti et al., 2011	Humans	Grafts from donors after brain death, including steatotic and non-steatotic liver grafts.	5 to 10 h	Not specified (liver samples collected before abdomen closure).	*Steatotic grafts* Autophagy markers: Expression of LC3 and Beclin-1. *Non-steatotic grafts* Autophagy markers: Rarely observed LC3 o Beclin-1 expression.	YES. Ischemic preconditioning.	*Steatotic grafts* Autophagy enhancement: ↑LC3, ↑Beclin-1. Liver damage: No changes in transaminases, ↓Acute rejection. *Non-steatotic grafts* No changes in autophagy parameters. Liver damage: ↓ALT and AST in non-steatotic grafts.

Investigations about the role of autophagy in clinical liver transplantation are very limited. Research conducted to date, indicates that in optimal and steatotic transplantation, there is usually a slight expression of autophagy markers (Degli Esposti et al., 2011; Ricca et al., 2015; Nakamura et al., 2018, 2019; Chen et al., 2019b). It is important to note that in steatotic grafts, published reports do not specify whether the donors presented any metabolic comorbidity such as insulin resistance or the MetS. Although no strategy to directly regulate autophagy has been applied yet in clinical practice, results suggest that steatotic and non-steatotic transplanted liver grafts may benefit from strategies promoting autophagy, such as ischemic postconditioning, ischemic preconditioning, and recipient treatment with antibiotics to modulate gut microbiota (Degli Esposti et al., 2011; Ricca et al., 2015). This indicates that the mechanisms underlying I/R injury and associated with autophagy that have been described at the experimental level, also seem to occur at the clinical level. It encourages the study of new experimental strategies that are capable of modulating autophagy to improve postsurgical outcomes in steatotic liver grafts that are also compromised by metabolic comorbidities, and that later can be translated to clinical practice.

### Autophagy in Livers Affected by Metabolic Diseases and Undergoing Surgery

To date, there are only studies on autophagy involvement in liver surgery in the setting of NAFLD or hyperglycemia. However, findings described in the present review make it highly feasible that autophagy is also playing a crucial role in the damage of livers affected by the other metabolic diseases and that are submitted to surgery. As will be seen below, reports remain very limited and denote a huge area of opportunity in the field of liver surgery with underlying pathological conditions.

Experimentally, modulation of autophagy through various strategies protects livers affected by NAFLD and subjected to I/R intrinsic to resections. Lithium chloride-induced autophagy via modulating both glycogen synthase kinase 3 beta (GSK3b) and extracellular signal-regulated kinase (ERK) 1/2 pathways was beneficial for steatotic livers undergoing I/R (Kan et al., 2017). A therapeutic effect was also achieved by treatment with exogenous hydrogen sulfide, which reduced ER stress and the class A scavenger receptor (SRA) pathway, and promoted autophagy. This led to the amelioration of hepatic damage by reducing oxidative stress and inflammation (Ruan et al., 2019). Ischemic preconditioning, a surgical therapeutical strategy consisting of a short period of ischemia followed by a brief period of reperfusion before a sustained ischemic insult, proved beneficial in a mouse model of I/R injury in steatotic livers, by increasing autophagic flux. This surgical technique restored mitochondrial function via heme oxygenase-1 (HO-1)-mediated autophagy and protected against damage (Liu et al., 2016). Such outcomes mean that pharmacological treatments that stimulate autophagy could be potentially useful in livers affected by metabolic diseases and that are subjected to surgery. Accordingly, another study revealed that calpain 2 inhibition enhanced autophagy, decreased mitochondrial dysfunction, suppressed cell death, and improved injury in livers affected by NAFLD and obesity and undergoing I/R (Zhao et al., 2016). However, it should be mentioned that opposite results have also been described in HFD-fed mice exposed to I/R, since treatment with Exendin 4, a glucagon-like peptide 1 analog, mitigated autophagy thus ameliorating hepatocellular injury, and preserving mitochondrial integrity (Gupta et al., 2014). These controversies could be caused by the great diversity of experimental conditions described in the literature in warm I/R models, such as different times of ischemia or reperfusion, induction of ischemia in different liver regions, or various ways of inducing steatosis. To reach a consensus on the most convenient way to modulate autophagy in liver surgery in the presence of NAFLD, the effects of I/R on autophagy occurring in each of these different experimental conditions must be characterized.

Very few experimental studies have evaluated the role of autophagy in transplantation of NAFLD grafts (Gracia-Sancho et al., 2013; Zaouali et al., 2013a, b, 2017; Panisello-Roselló et al., 2017, 2018; Zhang et al., 2019). To date, reported results differ depending on the experimental model used. Most of the studies on steatotic liver grafts have used an *ex vivo* reperfusion model. In these conditions, when autophagy parameters were analyzed after short reperfusion periods (1 to 2 h), results agreed with those reported in optimal liver grafts: (a) cold ischemia induces a mild increase or no change in autophagy parameters; and (b) grafts benefited from pharmacological strategies that increased autophagy activity (Gracia-Sancho et al., 2013; Zaouali et al., 2013b, 2017). However, results were altogether different in the only study conducted in an *in vivo* model, since autophagy parameters after a prolonged reperfusion period (6 h), increased as a result of I/R injury. Furthermore, under these conditions, the pharmacological inhibition of autophagy protected steatotic liver grafts. This study also generated another important result in the setting of transplantation: in steatotic grafts, berberine inhibited autophagy and protected the graft, but the same drug activated autophagy in optimal grafts and decreased liver injury (Lin et al., 2017; Zhang et al., 2019). This observation underscores the very different signaling mechanisms that act in optimal *vs*. steatotic grafts, despite being subjected to the same surgical maneuvers.

As to the role of autophagy among the mechanisms responsible for liver injury-induced hyperglycemia, reports in the literature are scarce. In a streptozotocin-induced hyperglycemic mouse model, hyperglycemia aggravated thioacetamide-induced acute liver injury. In this model, the inflammatory response was stimulated by promoting liver resident macrophage NLRP3 inflammasome activation and inhibiting AMPK/mTOR autophagy signaling pathways. Autophagy restoration by AMPK activation or mTOR knockdown inhibited NLRP3 inflammasome activation in Kupffer cells and decreased thioacetamide-induced acute liver injury in hyperglycemic mice (Wang Q. et al., 2020). Considering that the autophagy-NLRP3 inflammasome pathway is also involved in NAFLD (Wan et al., 2016), perhaps the regulation of this signaling pathway could be useful to protect against damage in liver surgery with other metabolic comorbidities in addition to hyperglycemia.

Liver dysfunctions in the immediate postoperative period of liver resection or transplantation, especially in livers affected by underlying metabolic conditions, are still an unsolved problem. As the prevalence of metabolic diseases such as NAFLD, MetS, insulin resistance, obesity, or diabetes is increasing worldwide, the development of therapeutic options in patients affected by these diseases and also undergo liver surgery are required. There is no doubt that regulation of autophagy improves warm I/R injury in optimal livers, but is uncertain whether these benefits are maintained when livers affected by metabolic diseases face I/R injury. The relevance of autophagy steatotic liver transplantation associated with other related metabolic comorbidities has not been investigated. To date, in studied models of steatotic liver transplantation, only some mention specifically that the experimental animals were obese aside from harboring fatty liver disease; and in all published studies, information on the parameters that would reflect the associated presence of insulin resistance or MetS in the animals is omitted. To the best of our knowledge, the role of autophagy in experimental conditions in which the donor has steatosis and diabetes has not been evaluated. Thus, in the coming years, research should be carried out on autophagy and its associated molecular mediators in livers undergoing surgery with one or more metabolic diseases, as it is what happens in the clinical practice. So, concerning laboratory approaches, to use experimental models the closest to clinical practice is the best to design new therapeutic strategies to be developed and successfully applied in patients.

The lack of studies describing how metabolic diseases affect post-operative results of liver surgery in the medium and long term is evident, and even less is known to what extent autophagy is relevant in this setting. Intensive research is also necessary to establish whether autophagy is involved in post-surgical events such as transient hyperglycemia and the development of MetS in liver transplantation recipients. This information will allow us to elucidate whether autophagy regulation could lead to the generation of effective therapeutic strategies that could improve the current results of transplantation and resection, not only in the immediate postoperative period but also in the long term.

### Future Perspectives on Research in Autophagy and I/R Injury, in Livers Affected by Metabolic Comorbidities

To date, no experimental or clinical investigations have been carried out, in which the fatty liver undergoing surgery is combined with the presence of obesity, insulin resistance, MetS, and/or hyperglycemia, nor on the effects of autophagy under these conditions. The combination of several of these conditions is commonplace in clinical practice, due to their high prevalence. In those multiple disease scenarios, autophagy most likely plays a predominant role since it contributes to the development of each of these pathological entities when manifested separately. There are even some mediators of autophagy that are common to hepatic ischemia, steatosis, obesity, MetS, and diabetes, such as ER stress, AMPK, MAPK, or oxidative stress. Importantly, it should be taken into account that the effectiveness of treatments could be different depending on the combination of metabolic comorbidities that occurs in each situation, given that a beneficial therapy for steatotic livers in presence of obesity or MetS, may not be effective in the case of steatotic livers also suffering diabetes. This hypothesis is originated based on existing reports showing that some therapies that have been described to reduce hepatic I/R injury exhibit different effectiveness depending on the underlying pathological condition in the liver. By way of example, therapies that have been found to reduce injury in steatotic livers do not offer any benefit in non-steatotic grafts (Álvarez-Mercado et al., 2019). Therefore, in order to consider the autophagy pathway as a possible therapeutic target in patients with several metabolic comorbidities and that are subjected to transplantation or resection, autophagic molecular signaling must first be exquisitely characterized in experimental or clinical models of fatty livers with obesity, diabetes, and/or MetS (separately or in different combinations of these pathologies).

Much work is required before experimental results can be translated into clinical practice, and the first important step is to make use of experimental models that best mimic clinical conditions. In several experimental models of hepatic steatosis used nowadays, insulin resistance does not always develop, and then insufficiently would reflect the pathogenesis of NAFLD in patients. There are several NAFLD animal models available, among which the most common are mice fed the MCD or HFD. The MCD diet causes hepatic steatosis, and body weight loss but no insulin resistance. The HFD causes obesity, hepatic steatosis with mild injury, and insulin resistance. Genetically deficient ob/ob or db/db mice or Zucker rats, develop obesity and steatosis, but in some cases, this does not occur automatically, and they often need to be fed either the MCD or the HFD (Adkins et al., 2013). Combinations of fat- and carbohydrate-rich dietary components have been used in rodents to mimic MetS. Among such diets, the “cafeteria” diet has been used to induce severe obesity with glucose intolerance and liver steatosis classifiable as NAFLD. After following the cafeteria diet for 8 and 15 weeks, insulin resistance and high plasma triglyceride levels are greater than in animals fed a traditional lard-based HFD (Parafati et al., 2015). It is important to characterize which metabolic comorbidities (obesity, MetS, diabetes, insulin resistance, fatty liver) are present or predominant in each experimental model, in order to determine with greater accuracy which combination of pathological entities to study when submitting a fatty liver obtained from these models to liver surgery. Another highly relevant consideration when selecting an experimental model is to evaluate whether if it mimics clinical liver surgery. Regarding liver resection, there is a paucity of research using experimental models that best mimic the ischemic times commonly used or including partial hepatectomy in combination with vascular occlusion. Post-operative outcomes after liver resection are influenced by the magnitude of I/R injury, but also by the regenerative response. Hepatic ischemia causes liver injury and impairs regeneration, and these phenomena are further compromised if livers with underlying diseases (steatosis, cirrhosis, diabetes, etc.) are resected and undergo I/R injury (McCormack et al., 2007). In terms of modeling liver transplantation, experimental studies have been carried out in the absence of brain death or cardiac death, which means in surgical conditions very different to those occurring in clinical practice. Brain death and cardiac death cause important hemodynamic changes, hypoperfusion in the mesenteric microcirculation and warm hepatic ischemia, which might result in significant modifications in the mediators generated in the liver, and the concomitant inflammation. This could alter the autophagy pathways, and have deleterious effects on liver grafts used for transplantation. It is necessary to include pathological liver grafts in experimental studies, since the success, dysfunction or loss of liver grafts may be affected by the presence of steatosis, diabetes, MetS, etc. in potential donors. These conditions should be considered in future research to establish how they influence autophagy and post-operative outcomes. Since the effectiveness of a therapeutic modality aimed at improving post-surgical outcomes in patients undergoing liver surgery could differ depending on the surgical conditions, the type and characteristics of the liver and other factors, the use of experimental models that reproduce as closely as possible real clinical conditions is key to the effective translation of laboratory results into the clinical realm.

Several molecular mediators of autophagy in the steatotic liver are key in hepatic I/R injury, which represents an area of opportunity to explore treatments for protecting livers affected by metabolic diseases such as NAFLD, and those undergoing surgery. AMPK and ER stress are closely related at the level of molecular signaling mechanisms associated with autophagy in NAFLD models (Homma and Fujii, 2020), as also occurs in I/R. In fact, activation of AMPK, as well as inhibition of ER stress through drugs, are beneficial to regulate autophagy and protect steatotic livers that undergo surgery (Carrasco-Chaumel et al., 2005; Zaouali et al., 2013b), but its usefulness at the clinical level has not yet been evaluated. These findings indicate that regulation of AMPK and ER stress could be promising therapies. On the other hand, although JNK and ghrelin are also mediators involved in autophagy in fatty liver and I/R, their pharmacological modulation in livers with NAFLD and undergoing surgery could lead to serious difficulties. In non-surgical NAFLD models, activation of JNK, or ghrelin is associated with activation of autophagy, which is beneficial (Singh et al., 2009; Mao et al., 2015). However, it has been widely demonstrated that JNK activation is implicated with steatotic liver damage in transplantation, and warm ischemia alone or combined with resection (Lehmann et al., 2003; Massip-Salcedo et al., 2006; Ben Mosbah et al., 2010; Shaker et al., 2016; Li S. et al., 2017); and, although pharmacological regulation of ghrelin has been scarcely explored in liver surgery, first published reports in this regard indicate that treatments increasing hepatic ghrelin levels are harmful in non-steatotic liver grafts from brain dead donor, which represents an experimental model very close to clinical practice (Álvarez-Mercado et al., 2019). Therefore, to design effective strategies for protecting livers with metabolic diseases and undergoing surgery, it is required to perform meticulous research on the regulation of molecular mediators involved in both hepatic autophagy and I/R, using experimental models that include NAFLD and even other metabolic diseases, and that should be studied in diverse variants of liver surgery.

When attempting to develop new therapeutic strategies, one should consider mediators that have been recently described to modulate autophagy in obesity, NAFLD, or diabetes, but not yet been studied in conditions in which one or more of these comorbidities are combined with hepatic surgery. Table 3 shows some examples of this type of mediator. However, we must consider that the regulation of autophagy could also cause serious side effects that would be a serious inconvenience in hepatic surgery. Recent studies have demonstrated that pharmacological upregulation of autophagy decreases hepatotoxicity and steatosis in NAFLD. However, further studies are needed before autophagy activation can be considered a viable treatment against I/R injury in steatotic livers affected by other metabolic comorbidities, because upregulation of autophagy in hepatic stellate cells has been shown to foment their activation, and consequently, initiate liver fibrosis. Inhibition of mTOR activity can induce autophagy, but also inhibits cell proliferation. Thus, in liver resection with vascular occlusion, cell proliferation is detrimental, since it precludes or impairs liver regeneration (Matter et al., 2014). The liver responds to some stressors through global activation of autophagy, causing lipid, protein, and organelle degradation; but in some instances, upregulation of autophagy as a protective mechanism against liver injury could only involve a specific form of autophagy, such as lipophagy. For instance, autophagy upregulated as the first line of defense against alcohol-induced toxicity in the liver, selectively targets mitochondria, and LD, while excluding cytosolic proteins and other organelles. Future efforts should focus on establishing how selective forms of autophagy can be individually modulated for therapeutic purposes (Singh and Cuervo, 2012).

**TABLE 3:** New promising autophagy regulators to be evaluated as therapeutical strategies in livers affected by metabolic diseases and submitted to surgery.

Study	Modulation of autophagy	Type of liver pathology	Animal species or cell culture	Experimental model used to induce hepatic metabolic diseases	Results from administration of exogenous regulator of autophagy vs. untreated groups
Lee et al., 2020	Iridoids of *Valeriana fauriei*	Steatotic cells	Huh7 cells	Treatment with oleic acid.	Autophagy enhancement: ↑LC3 I to LC3 II conversion, ↑Autophagic vacuoles. ↑Lipophagy: ↓Lipid droplets. ↓mTORC1, ↓ULK1 phosphorylated. Other cellular effects: ↓Lipid accumulation.
	*Valeriana fauriei* 70% ethanol extract	Fatty liver, obesity.	C57BL/6 J mice	High fat diet for 13 weeks.	Autophagy enhancement: ↑LC3 I to LC3 II conversion, ↑Autophagic vacuoles. ↓mTOR phosphorylated, ↓ULK1 phosphorylated. Other liver effects: ↓Lipid accumulation, ↓Lipogenesis-related genes.
Sinha et al., 2014	Caffeine	Steatotic cells	HepG2 cells	Treatment with oleic acid and palmitic acid.	Autophagy enhancement: ↑LC3 I to LC3 II conversion, ↑ATG7, ↑ATG5, ↑Beclin, ↓p62 ↑Autophagosome formation. ↓mTOR phosphorylated. Other cellular effects: ↑Lipid clearance.
	Caffeine	Fatty liver	C57BL6 mice	High fat diet for 8 weeks.	Autophagy enhancement: ↑LC3 I to LC3 II conversion, ↓p62. ↓mTOR phosphorylated. Other liver effects: ↓Lipid accumulation, ↑Lipid uptake in lysosomes, ↑Fatty acid β-oxidation
Kuo et al., 2020	Magnolol	Steatotic cells	HepG2 cells and C57BL/6 mice primary hepatocytes	Treatment with palmitic acid.	Autophagy enhancement: ↑LC3 I to LC3 II conversion, ↑ATG7, ↓p62. ↓mTOR phosphorylated. Other cellular effects: ↓Cellular triglycerides, ↓Lipogenesis, ↑Lipolysis, ↓Inflammation.
	Magnolol	Hypertriglyceri-demia	Male Wistar Rats	Tyloxapol	Autophagy enhancement: ↑LC3 I to LC3 II conversion, ↑ATG5-12, ↑ATG7, ↑Beclin-1, ↓p62. ↓mTOR phosphorylated. Other liver effects: ↓Oxidative stress, ↓Lipogenesis, ↑Lipolysis, ↓Inflammation.
Baldini et al., 2020	Silybin	Steatotic cells	Rat hepatoma FaO cells	Treatment with oleic acid and palmitic acid.	Autophagy inhibition: ↓LC3 I to LC3 II conversion. Other cellular effects: ↓Lipid droplet diameter.
Wang C. et al., 2017	TFEB agonists: Digoxin, Ikarugamycin or Aloxidine dihydrochloride.	Fatty liver, hyperglycemia, hyperinsulinemia.	C57BL/6J mice	High fat diet for 1 month.	Autophagy enhancement: ↓p62. Other metabolic and hepatic effects: ↓Circulating glucose, ↓Circulating insulin, ↓Liver steatosis.
Li X. et al., 2017	Pectic bee pollen polysaccharide	Steatotic cells with insulin resistance	HepG2 cells	Treatment with high glucose and oleic acid and palmitic acid.	Autophagy enhancement: ↑LC3 I to LC3 II conversion, ↓p62. Other cellular effects: ↓Insulin resistance.
	Pectic bee pollen polysaccharide	Fatty liver, type 2 diabetes.	C57BL/6J mice	High fat diet for 8 weeks.	Autophagy enhancement: ↑LC3 I to LC3 II conversion, ↓p62. ↓mTOR phosphorylated Other metabolic and hepatic effects: ↓Glucose intolerance, ↓Insulin resistance, ↓Liver steatosis, ↓AST, ↑Lipolysis.
Gong et al., 2016	Akebia saponin D	Steatotic cells	Buffalo rat liver (BRL) cells	Treatment with oleic acid.	Autophagy enhancement: ↓LC3 I to LC3 II conversion, ↓Beclin, ↓p62, ↑Autolysosomes. ↓mTOR phosphorylated. Other cellular effects: ↓Lipid droplets.
	Akebia saponin D	Fatty liver, insulin resistance.	Ob/ob mice	High fat diet.	Autophagy enhancement: ↓LC3 I to LC3 II conversion, ↓Beclin, ↓p62, ↑Autophagosomes. Other metabolic and hepatic effects: ↓Circulating Glucose, ↓Circulating Insulin, ↓Insulin resistance, ↓Liver steatosis, ↓ALT and AST, ↓Apoptosis, ↓Oxidative stress.
Parafati et al., 2015	Bergamot polyphenol fraction.	Metabolic syndrome: Fatty liver, Obesity, Hyperglycemia, Hypertriglyceridemia.	Rcc:Han WIST rats	Cafeteria diet (15% protein, 70% carbohydrates, 15% fat) for 13 weeks.	Autophagy enhancement: ↑LC3 I to LC3 II conversion, ↑Beclin-1, ↓p62. ↑Lipophagy: ↓Lipid droplets. Other metabolic and hepatic effects: ↓Circulating Glucose, ↓Circulating Triglycerides, ↓Liver steatosis.
Huang et al., 2017	Ginsenoside Rb2	Steatotic cells	HepG2 cells and C57BL mice primary hepatocytes.	Treatment with high glucose and oleic acid.	Autophagy enhancement: ↑LC3 I to LC3 II conversion, ↓p62. ↑Lipophagy: ↓Lipid droplets. ↑SIRT1
	Ginsenoside Rb2	Fatty liver, obesity, diabetes.	C57BL/KsJ-Lepdb (db/db) mice	N/A	Autophagy enhancement: ↑LC3 I to LC3 II conversion, ↓p62. ↑SIRT1, ↓mTOR phosphorylated. Other metabolic and hepatic effects: ↓Circulating Glucose, ↓Insulin resistance, ↓Liver steatosis, ↓ALT and AST.
Shao et al., 2018	Exenatide	Fatty liver, diabetes.	C57BL/6 mice	High fat diet for 10 weeks and treatment with streptozocin.	Autophagy enhancement: ↑LC3 I to LC3 II conversion, ↑Beclin, ↑Autophagosomes. ↑Mitophagy: ↑Parkin, ↑BNIP3L. Other metabolic and hepatic effects: ↓Circulating Glucose, ↓Liver steatosis, ↓ALT, ↓Oxidative stress.
Cui et al., 2020	Chronic intermittent hypobaric hypoxia	Metabolic syndrome: Fatty liver, Obesity, Hypertension, Hyperglycemia, Hypertriglyceridemia, Insulin resistance.	Sprague Dawley rats	High fat diet and water supplemented with fructose for 16 weeks.	Autophagy enhancement: ↓LC3 I to LC3 II conversion, ↓Beclin-1, ↓p62. ↓mTOR phosphorylated, ↓ER stress (GRP78, CHOP). Other metabolic and hepatic effects: ↓Circulating Glucose, ↓Circulating Triglycerides, ↓Insulin resistance, ↓Liver steatosis, ↓ALT and AST.

Recent findings on autophagy signaling pathways in NAFLD could also be useful in the development of markers for hepatic injury progression. In NAFLD, lipolysosomes appear to reflect the progressive impairment of lysosomal function, and particularly, the capability of lysosomal hydrolases to catabolize fat. This is supported by the correlation between an increase in the number of lipolysosomes and disease activity, in terms of necroinflammation. Upregulation of lysosomal genes such as cathepsin D, LAMP1, LAMP2, Niemann-Pick-type C1 (NPC1), vacuolar H + -ATPase, B2 subunit (ATP6V1B2), TFEB, suggests an increase in the overall lysosomal mass and could be interpreted as an attempt to counteract lysosomal dysfunction (Houben et al., 2017). Cathepsin D expression is greater in NAFLD patients compared with controls, and a direct correlation has been observed between cathepsin D expression and both NASH and fibrosis. Thus, molecular markers of lipophagy impairment could help to identify hepatic injury in patients on time (Carotti et al., 2020). Further studies are required to verify whether quantification of lipolysosomes could be a feasible and reproducible tool to assess the severity of NAFLD and its propensity to progress. The p62/SQSTM1 is essential for LC3B recruitment in LD in ethanol-induced lipophagy, while p62/SQSTM1 knockdown triggers the accumulation of triglycerides and cholesterol. Recent reports suggest that serum p62/SQSTM1 levels could also become a potential biomarker in the diagnosis of patients with steatosis and lobular inflammation (Kuo et al., 2020). These may all become very valuable non-invasive tools to evaluate liver injury in the immediate postoperative period in patients subjected to liver resection or transplantation.

## Conclusion

Due to the high prevalence of obesity, MetS, NAFLD, and diabetes, in the coming years, there may be a need to use liver grafts from donors with these metabolic diseases to reduce the waiting list for liver transplantation. In the same way, the prevalence of such diseases causes an increasing presence of such diseases in liver resections. Livers with underlying pathological conditions possess higher sensitivity to I/R injury inherent to transplantation and resection and are at high risk of morbidity or mortality in the immediate postoperative period. Therefore, there is a growing need to develop therapies to improve the postoperative results of livers affected by obesity, MetS, NAFLD, or diabetes that are subjected to surgery.

As described in this review, autophagy is closely involved in signaling pathways underlying effects of obesity, MetS, and obesity on the liver; and also is implicated as molecular mechanisms to NAFLD and the extent of the damage caused by I/R as well as in surgical outcomes. These characteristics make autophagy regulation the basis of promising treatments in patients suffering from the conditions aforementioned and submitted to liver surgery. Unfortunately, whether hepatic autophagy following warm or cold I/R protects or contributes to the damage is still unsolved since results are controversial. There are even controversies as to how the regulation of hepatic autophagia occurs in conditions of obesity, MetS, NAFLD, or diabetes. This lack of understanding hampers their translation from bench to bedside.

Therefore, it is mandatory to increase the knowledge about autophagic molecular signaling in experimental or clinical models of obesity, MetS, NAFLD, or diabetes subjected to resection or transplantation. In this last surgical situation, it is important to clarify the mechanisms taking place in the recipient and donor. It should be bearing in mind that in clinical practice the most common scenario is patients combining NAFLD, obesity, MetS, or diabetes, and for this reason, preclinical studies must use adequate experimental ischemia when evaluating the relevance of autophagy in the setting of I/R. This knowledge is the premise for finding not only therapeutic strategies to prevent damage in patients with these metabolic diseases who undergo surgery but also to find useful and non-invasive biomarkers to monitor the progression of liver damage in these conditions. These advances would undoubtedly improve the clinical prognosis of liver surgery in the coming years.

## Author Contributions

AÁ-M, CR-A, MM-C, and AC-C gathered the related literature, prepared the figures, and drafted the manuscript. CP and AC-R participated in the design of the review and drafted the manuscript. All authors read and approved the final manuscript.

## Conflict of Interest

The authors declare that the research was conducted in the absence of any commercial or financial relationships that could be construed as a potential conflict of interest.

## Abbreviations

ADP: adenosine diphosphate
AGE: advanced glycation end product
Akt: serine-threonine protein kinase Akt
AMPK: adenosine 5 ′ -monophosphate-activated protein kinase
ASNS: asparagine synthetase
Atg: autophagy gen
ATP: adenosine triphosphate
ATP6V1B2: vacuolar H + -ATPase, B2 subunit
BNIP3: BCL2 and adenovirus E1B 19-kDa-interacting protein 3
CRY1: cryptochrome 1
CXCR3: chemokine (C-X-C motif) receptor 3
DNA: desoxyribonucleic acid
ER: endoplasmic reticulum
ERK: extracellular signal-regulated kinase
FA: fatty acids
FGF21: fibroblast growth factor 21
FOXO: forkhead box O
GSK3b: glycogen synthase kinase 3 beta
HFD: high-fat diet
HOTAIR: homeobox (HOX) transcript antisense RNA
I/R: ischemia/reperfusion
IKK: inhibitor of NF-κB kinase
IL: interleukin
IRE1: inositol-requiring enzyme 1
IRF1: interferon regulatory factor 1
IRGM: immunity-related guanosine triphosphatase family M
IRS: insulin receptor substrate
JNK: c-Jun NH(2)-terminal kinase
LAMP: lysosome-associated membrane protein
LC3: microtubule-associated protein light-chain 3
LD: lipid droplets
lncRNAs: long-non-coding RNAs
MAPK: mitogen-activated protein kinase
MC3R: melanocortin 3 receptor
MCD: methionine/choline-deficient
MetS: metabolic sydrome
miR: microRNA
mTOR: mammalian target of rapamycin
mTORC1: mammalian target of rapamycin complex 1
NAD +: nicotinamide adenine dinucleotide
NAFLD: non-alcoholic fatty liver disease
NASH: non-alcoholic steatohepatitis
NF-kB: nuclear factor kappa B
NIX: BNIP3-like (BNIP3L), also known as NIX
NLRP3: nucleotide-binding oligomerization domain (NOD)-like receptor (NLR) family pyrin domain containing 3
NO: nitric oxide
NPC1: Niemann-Pick-type C1
PARP: poly (ADP-ribose) polymerase
PERK: protein kinase R (PKR)-like endoplasmic reticulum kinase
PGAM5: phosphoglycerate mutase family member 5
PI3K: phosphoinositide-3 kinase
PINK1: phosphatase and tensin homolog (PTEN)-induced putative kinase 1
PKC: protein kinase C
PLIN2: perilipin 2
PPAR-γ: peroxisome proliferator-activated receptor gamma
RAGE: receptor for AGE
RBP4: retinol binding protein 4
RNA: ribonucleic acid
ROS: reactive oxygen species
Rubicon: run domain beclin-1-interacting and cysteine-rich domain-containing protein
S6K1: ribosome S6 protein kinase 1
Sirt1: silencing information regulator 1
SOD: superoxide dismutase
SQSTM: p62/sequestosome
SRA: class A scavenger receptor
T2D: type 2 diabetes
TFEB: transcription factor EB
TNF-α: tumor necrosis factor alpha
UPR: unfolded protein response
Vps34: vacuolar protein sorting 34.
